# Genetic Diversity and Population Structure of Broomcorn Millet (*Panicum* *miliaceum* L.) Cultivars and Landraces in China Based on Microsatellite Markers

**DOI:** 10.3390/ijms17030370

**Published:** 2016-03-14

**Authors:** Minxuan Liu, Yue Xu, Jihong He, Shuang Zhang, Yinyue Wang, Ping Lu

**Affiliations:** 1 Institute of Crop Science, Chinese Academy of Agricultural Sciences, Beijing 100081, China; liuminxuan@caas.cn (M.L.); ZSCAAS@163.com (S.Z.); wangyinyue1989@126.com (Y.W.); 2 School of Life Science, Jilin University, Changchun 130012, China; xuyue@126.com; 3 Institute of Crop Science, Gansu Academy of Agricultural Sciences, Lanzhou 030000, China; hejihonggaas@163.com; 4 Faculty of Life Science, Jilin Agricultural University, Changchun 130118, China

**Keywords:** genetic diversity, population structure, SSR markers, *Panicum miliaceum* L. varieties

## Abstract

Broomcorn millet (*Panicum miliaceum* L.), one of the first domesticated crops, has been grown in Northern China for at least 10,000 years. The species is presently a minor crop, and evaluation of its genetic diversity has been very limited. In this study, we analyzed the genetic diversity of 88 accessions of broomcorn millet collected from various provinces of China. Amplification with 67 simple sequence repeat (SSR) primers revealed moderate levels of diversity in the investigated accessions. A total of 179 alleles were detected, with an average of 2.7 alleles per locus. Polymorphism information content and expected heterozygosity ranged from 0.043 to 0.729 (mean = 0.376) and 0.045 to 0.771 (mean = 0.445), respectively. Cluster analysis based on the unweighted pair group method of mathematical averages separated the 88 accessions into four groups at a genetic similarity level of 0.633. A genetic structure assay indicated a close correlation between geographical regions and genetic diversity. The uncovered information will be valuable for defining gene pools and developing breeding programs for broomcorn millet. Furthermore, the millet-specific SSR markers developed in this study should serve as useful tools for assessment of genetic diversity and elucidation of population structure in broomcorn millet.

## 1. Introduction

Broomcorn millet (*Panicum miliaceum* L. (Poaceae); 2*n* = 4*x* = 36) is an annual warm season crop also known as proso, hog, white, yellow, or common millet [1]. One of the most ancient grain crops, its agricultural use in North China pushed back to the Pleistocene–Holocene boundary [2]. Broomcorn millet is cultivated widely across China; the main production area is along the Great Wall, where it serves as an important staple food [3]. The species is also planted for human and avian consumption in central Europe, Russia, India, Pakistan, Korea, Japan, and other parts of Eurasia [4], and has emerged as one of the most aggressive grass weeds in North America and Canada [5]. Broomcorn millet has the shortest growing cycle of any cereal, reaching maturity 60–90 days after sowing. The crop also has low water and nutrient requirements, allowing it to be cultivated at a wide range of altitudes, even on marginal agricultural land where other cereals do not succeed [6]. Broomcorn millet is also a health food because of its unique nutritional benefits: it features protein contents, especially those of alkaline ones, which are higher than levels in crops such as wheat, rice, and oats, an abundance of easily absorbed amino acids, and a relatively balanced array of trace elements and vitamin precursors [7]. For these reasons, broomcorn millet continues to be an important component of the Chinese diet.

The collection, evaluation, conservation, and utilization of crop germplasm have become one of the top agricultural research priorities in China [8]. Interest in the genetic diversity and structure of natural populations has increased because of the need to broaden knowledge of genetic variation in cultivated species [9]. A detailed understanding of genetic relationships among germplasm resources is vital for future breeding process like yield, quality, and resistance (including pest and disease) [10]. In addition, a thorough dig and research of germplasm conserved in gene bank can facilitate the introgression of useful gene into the existing commercial crop genetic base [11]. According to the differences in morphological traits, isozymes, DNA markers, as well as pedigree information and geographic origins, crop genetic diversity and relationship can be evaluated. Compared with restriction fragment length polymorphism (RFLP), amplified fragment length polymorphism (AFLP), and random amplification of polymorphic DNA (RAPD) markers, simple sequence repeats (SSRs) have been shown to produce higher levels of polymorphisms and to have much greater ability to identify unique alleles in crop germplasm [12]. SSRs constitute a superior molecular marker system, offering the advantages of being codominant, abundant, highly reproducible, highly polymorphic, and easy to assay. SSRs have been used to study genetic diversity in various crop species, including maize [13], soybean [14], sorghum [15], cowpea [16], and foxtail millet [17]. SSRs have also been used to construct linkage maps, assess phylogenetic and population genetic relationships, and identify molecular markers for marker-assisted selection [18].

More than 8700 accessions (landraces and varieties) of *P. miliaceum* (*Panicum miliaceum*) are conserved in the National Gene Bank of the Institute of Crop Science, Chinese Academy of Agricultural Sciences (Beijing, China). Although abundant morphological variation exists within the broomcorn millet accessions, assessment of their genetic diversity using DNA markers has been inadequate. Previous analyses of genetic variation in *P. miliaceum* have employed isozymes [19], RAPDs [20], AFLPs [21], and SSRs transferred from other cereal species [22], as well as markers developed in broomcorn millet by *de novo* methods [23]. The resulting data are limited, however, and cannot fully reveal genetic relationships among accessions. Furthermore, no research has been performed on the genetic diversity and inter-relationships of cultivated varieties of broomcorn millet in China.

In this study, millet-specific SSR primers developed in our laboratory by high-throughput sequencing were used to identify polymorphisms and to analyze the genetic diversity and structure of 88 accessions comprising 56 main varieties cultivated in China and 32 parental lines.

## 2. Results

### 2.1. SSR Polymorphic Variation

Using the 67 SSR primer pairs that produced clear polymorphic fragments among eight representatives during preliminary screening, we detected 179 alleles and 349 genotypes in the 88 studied accessions. Details of uncovered polymorphism levels and other parameters are given in Table 1. Observed number of alleles (Na), is one of the most important indexes of genetic differentiation associated with populations, types, and geographical sites [17]. Among the 88 accessions, Na per locus varied from 2 to 5, with a mean value of 2.7, and the number of amplified genotypes varied from 3 to 15, with an average of 5.2. The effective number of alleles (Ne) for each locus varied between 1.05 and 4.29, with an average of 1.995 per locus. Of 179 alleles, 10 (5.59%) were rare, with a frequency less than 0.05 in the entire set of samples. Approximately 50% and 32% of polymorphic SSR loci were associated with two and three alleles, respectively (Table 2). Values of Shannon's information index (*I*) varied from 0.1085 to 1.5194 per locus, with an average of 0.7254, while expected heterozygosity (He) and observed heterozygosity (Ho) ranged from 0.0447 to 0.7713 (mean = 0.4447) and 0 to 0.9545 (mean = 0.2348), respectively. Some loci, such as F786, F1036, F1067, F1071, F2185, BM306, and BM344, had a Ho of 0, suggesting universal outcrossing between individuals or perhaps between wild populations and nearby cultivated broomcorn millet. The value of genetic diversity which calculated according to Nei’s 1973 (*H*) ranged from 0.0444 (for F1036) to 0.7669 (for F1380), with an average of 0.4419. With respect to *F*_ST_, an index of genetic differentiation or the genetic distance between wild and cultivated accessions, values of the 67 applied markers ranged widely: from 0.0434 (BM114) to 0.8342 (F1071), with a mean of 0.2988. Polymorphism information content (PIC) values for each SSR ranged from 0.0434 (F1036) to 0.7288 (F1380), with an average of 0.376, indicating a moderate level of genetic diversity in Chinese broomcorn millet. In the analyzed samples, values of Na and Ne per locus were most strongly correlated with PIC (*r* = 0.966–0.993, *p* < 0.05), followed by *I*, He, and *H*.

### 2.2. Comparative Genetic Diversity of Broomcorn Millet Varieties from Different Populations

As evidenced by the estimates of population genetic diversity listed in Table 3, genetic differences existed among the 88 accessions derived from the 11 populations of five ecotypes. We detected 1420 alleles at 67 SSR loci in the 88 accessions, with the total number of alleles in each population ranging from 94 to 167 (Table 4). A total of 240 alleles were fixed among the 11 populations (Table 4), with the highest percentages of fixed alleles 64.2%, 56.7%, and 46.3% appearing in population 9, 7, and 2, respectively. Ap ranged from 38.84% to 95.52%, with a mean of 67.98% between populations, while Na per population ranged from 1.516 to 2.493 and averaged 1.980 (Table 3). Ho in each population varied from 0.197 to 0.33, with an average of 0.236. Average *I*, *H*, and PIC per locus varied among populations from 0.318 to 0.619 (average = 0.497), 0.215–0.427 (average = 0.310), and 0.345–0.420 (average = 0.380), respectively. The lowest genetic parameter values were found in accessions constituting population 9; this finding implies that the accessions from population 9 were very closely related, with a majority of loci (64.2%) observed to be fixed (Table 4). Accessions of population 8 which, from Inner Mongolia, China, exhibited the highest genetic diversity, displaying the highest values of all genetic parameters except for PIC.

### 2.3. Genetic Relationships Based on Cluster Analysis

**Figure 1:**
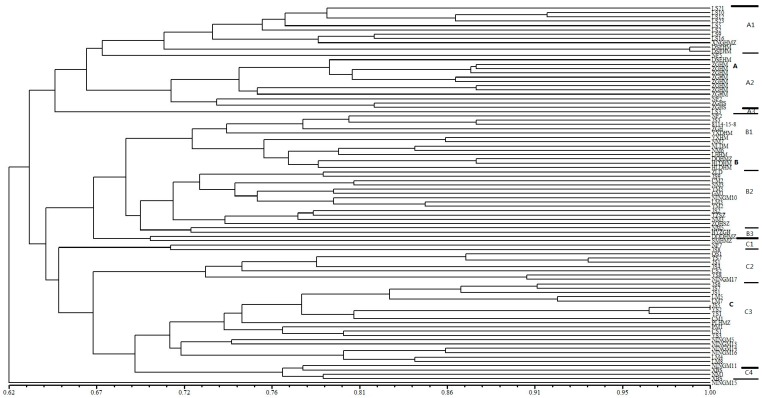
Dendrogram generated by UPGMA cluster analysis of 88 broomcorn millet accessions based on data from 67 SSR markers. A, B, C are main groups by cluster, A1, A2, A3, B1, B2, B3, C1, C2, C3, C4 are subgroups in each main group. Thick line is used to divide main group and thin line is used to divide subgroup.

Unweighted pair-group method with arithmetic (UPGMA) cluster analysis based on genetic similarity values among the 88 broomcorn millet accessions yielded the dendrogram shown in Figure 1. As seen in the dendrogram, the most genetically similar accessions were two samples from Inner Mongolia bearing the same name: Dongsheng Erhuangmi. The two most divergent accessions were Longshu3 from Heilongjiang and Ningmi15 from Ningxia, China. The cluster analysis divided the 88 accessions into four discrete groups at a genetic similarity value of 0.633 (Figure 1). Each group included accessions from at least one province, with each province represented in one to three groups (Table 5). Group A contained 25 accessions, including a series of Longshu varieties and their parents from Heilongjiang Province, China and 11 accessions from Inner Mongolia, China. This group was further subdivided in subgroups A1 (Heilongjiang), A2 (Inner Mongolia), and A3. Group B comprised 29 accessions: 16 of the 23 varieties collected from Inner Mongolia, four from Shanxi, China (Jinshu2, TianzhenShuzi, Jinshu9, and Ziluodai), three from Ningxia, China (Ningmi10, Ziganhong, and HaiyuanZiganhong), and two each from Heilongjiang (Nianfeng2 and Longshu3), Gansu, China, (Ganmi1 and Longmi3), and Shaanxi, China (Shenmuhongmizi and Yumi2). This group was further divided in three subgroups, of which B1 mainly included varieties and parents from Inner Mongolia. Group C consisted of 33 accessions: 11 of the 15 samples from Shanxi, seven from Ningxia, five from Inner Mongolia, four from Gansu, and two each from Jilin, Shaanxi, and Heilongjiang. Group C was separated into four subgroups, with C2 and C4 mainly comprising varieties from Shanxi and Inner Mongolia, respectively. Group D consisted of only one accession, Ningmi15 from Ningxia. This grouping of accessions based on polymorphic SSR loci is consistent with the geographic source and genetic background of the analyzed samples. These results also indicate that the breeding of broomcorn millet in the different provinces has proceeded in isolation.

### 2.4. Population Genetic Structure of Chinese Broomcorn Millet Varieties

We evaluated population structure and differentiation of the 88 accessions from different provinces using a Bayesian Markov chain Monte Carlo approach as implemented in STRUCTURE 2.2.3.

**Figure 2:**
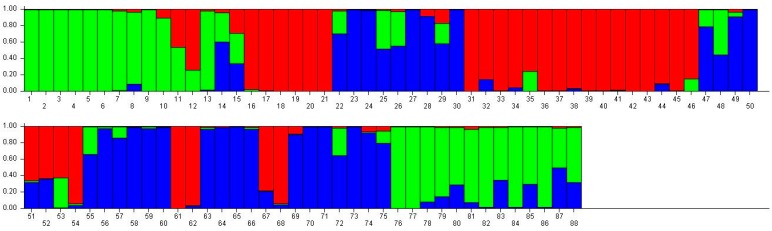
Population structure of 88 broomcorn milletcultivars based on STRUCTUREanalysis at *K* = 3. Different colors represent different groups and a bar represents a sample. The proportion of a color in a bar which is higher than other colors indicates that this sample belongs to the group which the color represents. 1–15 samples come from Heilongjiang (1, Longshu21; 2, Longshu5; 3, Longshu10; 4, Longshu9; 5, Longshu23; 6, Xiaonangouheimizi; 7, Longshu12; 8, LOngshu3; 9, Longshu16; 10, Longshu2; 11, NIanfeng5; 12, Nianfeng2; 13, Nianfeng2-1; 14, Qishu1; 15, Nianfeng7), 16–30 samples come from Shanxi (16, Jinshu3; 17, Ziluodai; 18, Jinshu2; 19, Tianzhenshuzi; 20, Jinshu9; 21, 8114-15-8; 22, Yanshu7; 23, JInshu6; 24, Yanshu8; 25, Jinshu8; 26, Jinshu4; 27, Jinshu5; 28, Jinshu7; 29, Pinmi1; 30, Jinshu1), 31–50 samples come from Inner Mongolia (31, Yixuanhuangmi; 32, Zhunqihuangshuzi; 33, Neimi5; 34, Neimi3; 35, Yixuandahongmi; 36, Chimi2; 37, Yimi5; 38, Niuluandanmi; 39, Neimi7; 40, Linhehuangmi; 41, Neimi6; 42, Daqiqingmizi; 43, Helindahuangmi; 44, Helingdahuangmi2; 45, Neimi3; 46, Dangdidahuangmizi; 47, Chishu1; 48, Chishu2; 49, Chimi1; 50, Neimi1), 51–60 samples come from Ningxia (51, Ningmi10; 52, Haiyuanziganhong; 53, Ningmi15; 54, Ziganhong; 55, NIngmi5; 56, Ningmi11; 57, Ningmi13; 58, Ningmi14; 59, Ningmi16; 60, Ningmi17), 61–66 samples come from Gansu (61, Longmi3; 62, Ganmi1; 63, Longmi4; 64, Longmi5; 65, Longmi7; 66, Longmi8), 67–71 samples come from Shaanxi (67,Yumi2; 68, Shenmuhongmizi; 69, Yushu3; 70, Yushu2; 71, Yushu1), 72–73 samples come from Jilin (72, Jiushu1; 73, Panlonghuangmi), 74–88 are samples come from Inner Mongolia which with same name (74–75 are Xiaobaishu; 76–78 are Dongshengerhuangmi; 79–88 are Ziganhongmi).

**Figure 3:**
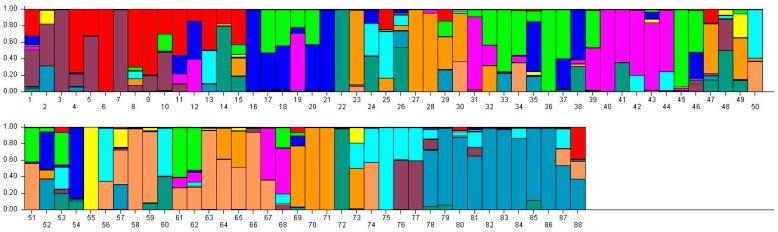
Population structure of 88 broomcorn millet cultivars based on STRUCTURE analysis at *K* = 11. Different colors represent different groups and a bar represents a sample. The proportion of a color in which a bar is higher than other colors indicates that this sample belongs to the group which the color represents. 1–15 samples come from Heilongjiang (1, Longshu21; 2, Longshu5; 3, Longshu10; 4, Longshu9; 5, Longshu23; 6, Xiaonangouheimizi; 7, Longshu12; 8, LOngshu3; 9,Longshu16; 10, Longshu2; 11, NIanfeng5; 12, Nianfeng2; 13, Nianfeng2-1; 14, Qishu1; 15, Nianfeng7), 16–30samples come from Shanxi (16, Jinshu3; 17, Ziluodai; 18, Jinshu2; 19, Tianzhenshuzi; 20, Jinshu9; 21, 8114-15-8; 22, Yanshu7; 23, JInshu6; 24, Yanshu8; 25, Jinshu8; 26, Jinshu4; 27, Jinshu5; 28, Jinshu7; 29, Pinmi1; 30, Jinshu1), 31–50 samples come from Inner Mongolia (31, Yixuanhuangmi; 32, Zhunqihuangshuzi; 33, Neimi5; 34, Neimi3; 35, Yixuandahongmi; 36, Chimi2; 37, Yimi5; 38, Niuluandanmi; 39, Neimi7; 40, Linhehuangmi; 41, Neimi6; 42, Daqiqingmizi; 43, Helindahuangmi; 44, Helingdahuangmi2; 45, Neimi3; 46, Dangdidahuangmizi; 47, Chishu1; 48, Chishu2; 49, Chimi1; 50, Neimi1), 51–60 samples come from Ningxia (51, Ningmi10; 52, Haiyuanziganhong; 53, Ningmi15; 54, Ziganhong; 55, NIngmi5; 56, Ningmi11; 57, Ningmi13; 58, Ningmi14; 59, Ningmi16; 60, Ningmi17), 61–66 samples come from Gansu (61, Longmi3; 62, Ganmi1; 63, Longmi4; 64, Longmi5; 65, Longmi7; 66, Longmi8), 67–71 samples come from Shaanxi (67,Yumi2; 68, Shenmuhongmizi; 69, Yushu3; 70, Yushu2; 71, Yushu1), 72–73 samples come from Jilin (72, Jiushu1; 73, Panlonghuangmi), 74–88 are samples come from Inner Mongolia which with same name (74–75 are Xiaobaishu; 76–78 are Dongshengerhuangmi; 79–88 are Ziganhongmi).

Since the number of genetic groups (*K*) showed clear peaks at 3 and 11, so we analyzed the genetic structure of the 88 accessions separately for these two values of *K*. We think that may be better for analysis of population structure of tested samples. At *K* = 3, three main groups could be distinguished (Table 6). Group 1 consisted of 31 accessions, all from Mongolian plateau (16) and the Loess Plateau (six from Shanxi, four from Ningxia, two from Gansu, and two from Shaanxi), except for Nianfeng2 from Heilongjiang. Group 2 consisted of 26 accessions, all from Northeast (13 accessions from Heilongjiang) and Mongolian plateau (12), except for Ziganhongshu from Shaanxi. Group 3 comprised 31 accessions; 25 were from the Loess Plateau (ninie from Shanxi, six from Ningxia, four from Gansu, and four from Shaanxi), with the remaining accessions from Mongolian plateau (five) and Northeast (two from Jilin, and one from Heilongjiang). At *K* = 11, the 88 accessions were divided into 11 groups (Table 7). With the exception of Dongsheng Erhuangmi in Group 8 from Inner Mongolia, Group 1 and Group 8 accessions all came from Heilongjiang Province; these two groups were therefore considered to be representative of the Northeast gene pool. Most accessions from groups 2 (seven accessions), 5 (eight accessions), and 10 (nine accessions) were from Inner Mongolia and, thus, constituted the Mongolian plateau gene pool. Most accessions in groups 3 (five accessions), 7 (five accessions), and 11 (three accessions) were collected from Shanxi Province, therefore representing the Loess Plateau and Alpine region gene pools. Most accessions in Group 9 were from Ningxia (four accessions) and Gansu (four accessions); this group, therefore, corresponded to the Northwest gene pool. The accessions were colored according to their STRUCTURE assignments at *K* = 3 (Figure 2) and *K* = 11 (Figure 3). These results closely mirrored the pattern of diversity revealed by the UPGMA dendrogram.

## 3. Discussion

### 3.1. Genetic Diversity and Population Structure of Broomcorn Millet in China

Broomcorn millet, one of the most ancient drought- and salt-resistant cereal crops [24,25,26] with an extremely short ripening time, is extensively cultivated for food and fodder in China, India, Russia, Central Europe, the Middle East, and North America [4]. As recorded in descriptions and data standards for broomcorn millet [27], the crop shows a high degree of variation in morphological features such as seed color (white, gray, yellow, red, brown, black, or compound), panicle type (lateral-or dense-panicled), inflorescence color (green or purple), and grain number per spikelet (one to three) across its distributional range. Although subspecies, races, and biotypes of broomcorn millet have been proposed [28], the races may not have eco-geographic unity, and weedy and wild types are often indistinguishable from cultivated varieties. Attempts to use isozymes and protein markers to distinguish the interspecies have not been successful [19,20]. Although molecular markers, such as RAPDs [20], AFLPs [21], inter-simple sequence repeats [6], single nucleotide polymorphisms [6], and SSRs [4,22,23] have been used to study broomcorn millet and its relatives, different conclusions have been obtained regarding its genetic diversity. M’Ribu and Hilu [20] used RAPDs to assess variation in four *Panicum* species and broomcorn millet; they found that broomcorn millet accessions exhibited high polymorphism levels and grouped together according to their geographical regions of origin. Conversely, Karam [21] compared the genetic diversity of three domestic and nine wild broomcorn millet biotypes from the United State and Canada; in that study, estimated genetic distances among biotypes ranged from 0.02 to 0.04, and a UPGMA cluster analysis revealed two distinct groups with no geographic association. These conflicting results may be due to the use of samples of different origins or unsuitable molecular markers. In our study, we analyzed 88 broomcorn millet accessions, including 56 cultivated varieties and 32 landraces, with several of the latter being parents of some varieties. The 67 SSR primers in our study possessed an average of 2.71 alleles per locus, and the value of PIC and He was 0.376 and 0.445, respectively. These important genetic parameters are higher than those reported for broomcorn millet from other countries based on SSR [23] or RAPD [20] markers, but lower than those uncovered in Chinese landraces [22]. The genetic diversity of China broomcorn millet thus appears to be much richer than that of other countries. In the UPGMA analysis in our study, cultivated varieties were grouped according to the geographical regions in which they were registered, with specific varieties and their parents often placed in the same group. This result is in accordance with that of most previous research [20,22,23], and indicates the existence of extensive genetic variation within different ecological growth areas and complex genetic relationships between various populations of broomcorn millet. The isolated position of Ningmi15 (from Ningxia Province) in the dendrogram is probably due to a high number of missing data points.

The observed association of varieties from contiguous regions such as Gansu and Ningxia has several possible explanations, such as similar natural conditions, artificial selection within the two regions, or seed movement and gene flow [20]. On the basis of their mixed genetic structures, most of the varieties tested in this study seem to be derived from hybridization events. For example, evidence of hybridization can be discerned for Longshu23 (accession 5 in Figure 3, with male parent XiaonangouHeimizi and female parent Longshu12), Jinshu9 (accession 20, with 8114-15-8 as one parent), Neimi5 (accession 33), and Neimi7 (accession 39, with parents LinheHuangmi and ZhunqiHuangshuzi).The results of cluster and genetic structure analysis revealed by our study will be valuable for defining gene pools and developing breeding programs for broomcorn millet. Breeders can select suitable accessions in their own ecotype to cross according to the dendrogram result.

### 3.2. SSRs as Effective Molecular Markers for Genetic Diversity Assessment of Broomcorn Millet

Compared with many other molecular markers, SSRs have several advantages: they are abundant and highly polymorphic, codominantly inherited, analytically simple, and readily transferable [29]. SSRs have been widely used to analyze the genetic diversity of various crop species. The first application of SSRs to broomcorn millet was by Hu *et al.* [22], who selected 983 SSR primers, including 450 from rice, 380 from wheat, 115 from oat, and 38 from barley to evaluate the genetic diversity of 118 broomcorn millet accessions. Although their study revealed a high level of genetic diversity, applicability of the SSR markers transferred from other crops was low: only 46 (4.6%) of the 983 primers generated clear and reproducible polymorphic fragments. To effectively evaluate the genetic diversity of broomcorn millet, additional millet-specific markers are therefore needed. Recent advances in library enrichment techniques and automated sequencing have simplified and accelerated the development of SSR markers in a cost-effective way [23]. As a result, species-specific SSR markers for various crops, such as foxtail millet [30], oats [31], faba bean [32], and grasspea [33], have been developed and characterized for future studies. Cho *et al.* [23] developed and characterized 25 polymorphic SSR markers for broomcorn millet through construction of an SSR-enriched library obtained from genomic DNA. Nevertheless, the number of polymorphic SSRs developed in that study was insufficient for the evaluation of the more than 8800 accessions of broomcorn millet conserved in the National Center for Crop Germplasm Conservation of China. We, therefore, used high-throughput sequencing to develop 500 SSR primer pairs in our laboratory and screened them for polymorphisms using eight representatives randomly selected from 88 accessions. Of the 500 pairs, 162 (32.4%) produced clear, reproducible, polymorphic fragments; 67 (13.4%) were additionally found to be polymorphic in more than 80% of accessions. After comparing our results with previous research [22], we conclude that the millet-specific SSR markers developed in this study have higher efficiency than SSRs transferred from other crops; they should serve as useful tools for the assessment of genetic diversity and the elucidation of population structure in broomcorn millet.

### 3.3. Origin and Evolution of Broomcorn Millet

Although Central Asia, China, and Central Europe have all been proposed as the specific area of domestication of broomcorn millet and its wild ancestors, the original location has not been definitively determined [34]. In addition, *in situ* hybridization has suggested that witch grass (*P. capillare* L.), a weedy, diploid (2*n* = 18) New World species, may be an ancestor of broomcorn millet [34]. Analysis of genetic diversity can provide insights into the origin and evolution of broomcorn millet. Our study revealed that Chinese accessions are typically more genetically diverse than those of other countries, a result consistent with the findings of Hu *et al.* [22]. Those authors also observed that genetic similarity coefficients of Loess Plateau ecotype accessions were significantly lower than those of other ecotypes, suggesting that the Loess Plateau is the original site of *P. miliaceum*. In our study, the genetic diversity of accessions from the Mongolian Plateau was slightly higher than that of other ecotypes, perhaps because of the effects of breeding programs or other reasons requiring further investigation. 

## 4. Materials and Methods

### 4.1. Plant Materials

A total of 88 broomcorn millet accessions (56 varieties and 32 parents) collected from seven main millet-producing Chinese provinces were provided by the institutions listed in Table S1.These accessions were divided into 11 populations according to sources. Populations 8–11 are four landrace populations, with all accessions from a given population having the same name. As indicated in Table S1, the 88 accessions belonged to five different ecotypes: Northeast (20 accessions), Loess Plateau (17), Mongolian Plateau (29), Northwest (16), and Alpine Region (6). Prior to experimental use, all plant materials were reproduced for three generations through strict self-crossing.

### 4.2. DNA Isolation

Seeds of each accession were sown in plastic pots (10 cm diameter) and grown under greenhouse conditions. Total genomic DNA was extracted from young leaves of 15–20-day old seedlings based on the modified cetyltrimethylammonium bromide method described by Edward *et al.* [35]. The relative purity and concentration of extracted DNA was evaluated on a Nano Drop ND-1000 instrument (NanoDrop, Wilmington, DE, USA). The final concentration of each DNA sample was adjusted to 30 ng·µL^−1^.

### 4.3. Primer Screening and Microsatellite Amplification

We used 500 pairs of SSR primers (Table S2.) developed in our laboratory by high-throughput sequencing to identify polymorphisms in eight representatives randomly selected from the 73 non-repeated accessions. All primers were synthesized by Dingguo Gene Co. (Beijing, China). A total of 162 primer pairs producing clear and reproducible polymorphic fragments among the eight accessions were used in further tests to assess the genetic diversity of all 88 accessions.

Polymerase chain reaction (PCR) amplifications were performed in 10 µL volumes containing 1.6 µL of 10× PCR buffer (containing 20 mM·Mg^2+^), 0.2 µL of each 10 mM dNTP, 0.1 µL of 5 U·µL^−1^ *Taq* DNA polymerase, 0.5 µL of a 5 µM solution of each primer, 1 µL of 30 ng·µL^−1^ genomic DNA, and 6.1 µL of ddH_2_O. Reactions were carried out in a PTC-100 Thermo-Cycler (ALT INC., East Lyme, CT, USA) using the program as follows: (1) initial denaturation at 94 °C for 5 min; (2) 39 cycles of denaturation at 94 °C for 45 s; (3) annealing at 55 °C for 50 s;(4) extension at 72 °C for 1 min;(5) a final extension at 72 °C for 10 min. The PCR-amplified products were resolved by 8% polyacrylamide gel electrophoresis, with DNA bands visualized by silver nitrate staining. Allele sizes were determined using a 50-bp DNA ladder (Tiangen, Beijing, China).

### 4.4. Data Analysis

Allele presence and absence was scored for each SSR marker as 1 and 0, respectively. These scores were stored in an Excel file as a binary matrix and served as the basis of the genetic diversity analysis.

POPGENE 1.31 [36] was used to calculate the following measures of genetic diversity: observed number of alleles (Na), effective number of alleles (Ne), observed heterozygosity (Ho), expected heterozygosity (He), Nei’s gene diversity (*H*) [37], and the Shannon–Weaver index (*I*). Geographical differentiation was evaluated by estimating *F*-statistic (*F*_ST_) values among geographical regions using POPGENE. The Simpson diversity index for each SSR, also known as the polymorphism information content (PIC), was calculated using the program PIC-CALC 0.6. Using a similarity matrix generated from the proportion of shared fragments [38], genetic relationships among genotypes were determined by cluster analysis based on the unweighted pair group method of mathematical averages (UPGMA) as implemented in NTSYS2.1. We used STRUCTURE version 2.3.4 to identify genetic groups within the 88 broomcorn millet varieties and their parents. STRUCTURE analysis is a Bayesian approach that uses no *a priori* classification and divides samples into *K* populations according to the allele frequencies at each locus. The most likely number of genetic groups (*K* = 1 to 10) was estimated following the procedure of Evanno *et al.* [39], who proposed the *ad hoc* statistic Δ*K*. Program settings included admixture ancestry and correlated marker frequency models, with α inferred from the data and lambda set to 1 [39]. Twenty independent Markov chain Monte Carlo runs, each consisting of 1,000,000 iterations with a burn-in of 500,000 iterations, were carried out for each *K.*

## 5. Conclusions

In conclusion, our data indicates there have abundant genetic variation within different ecological growth areas and complex genetic relationships between various populations of broomcorn millet. On the other hand, the millet-specific SSR markers developed in this study can be served as effective molecular tools for the assessment of genetic diversity and the elucidation of population structure in broomcorn millet.

## Figures and Tables

**Table 1:** Genetic parameters of the 67 polymorphic simple sequence repeat markers used in this study.

Locus Name	Ng ^a^	Na ^b^	Ne ^c^	*I* ^d^	Ho ^e^	He ^f^	*H* ^g^	*F*_ST_^h^	PIC ^i^
F265	10	4	3.215	1.225	0.830	0.693	0.689	0.121	0.626
F258	6	3	2.997	1.098	0.435	0.670	0.666	0.150	0.592
F334	3	2	1.984	0.689	0.136	0.499	0.496	0.449	0.373
F503	10	4	3.350	1.289	0.288	0.706	0.702	0.209	0.649
F510	3	2	2.000	0.693	0.026	0.503	0.500	0.505	0.375
F515	6	3	1.440	0.585	0.034	0.307	0.305	0.408	0.284
F619	3	2	1.585	0.556	0.102	0.371	0.369	0.207	0.301
F621	3	2	1.512	0.522	0.091	0.341	0.339	0.248	0.281
F630	3	2	1.527	0.529	0.080	0.347	0.345	0.252	0.285
F632	6	3	2.281	0.952	0.552	0.565	0.562	0.175	0.500
F691	3	2	1.996	0.692	0.091	0.502	0.499	0.352	0.374
F1080	6	3	1.586	0.682	0.023	0.372	0.370	0.206	0.339
F653	10	4	2.400	1.005	1.000	0.589	0.583	0.051	0.498
F1761	3	2	1.994	0.692	0.057	0.501	0.498	0.296	0.374
F1036	3	2	1.047	0.109	0.000	0.045	0.044	0.123	0.043
F1065	10	4	2.692	1.180	0.322	0.632	0.629	0.200	0.585
F1429	10	4	3.665	1.340	0.955	0.731	0.727	0.079	0.677
F1400	6	3	1.990	0.830	0.205	0.500	0.498	0.423	0.427
F1387	10	4	3.712	1.346	0.886	0.735	0.731	0.139	0.681
F1380	15	5	4.290	1.519	1.000	0.771	0.767	0.155	0.729
F635	6	3	1.444	0.574	0.109	0.310	0.307	0.726	0.281
F746	6	3	1.456	0.594	0.114	0.315	0.313	0.449	0.289
F780	3	2	1.831	0.646	0.125	0.458	0.454	0.404	0.351
F836	10	4	1.413	0.627	0.094	0.295	0.292	0.803	0.280
F845	6	3	1.630	0.692	0.046	0.389	0.387	0.274	0.347
F850	6	3	1.399	0.539	0.034	0.287	0.285	0.226	0.261
F1067	6	3	1.147	0.280	0.000	0.129	0.128	0.273	0.123
F1071	6	3	1.135	0.269	0.000	0.120	0.119	0.834	0.114
F1553	3	2	1.585	0.556	0.057	0.371	0.369	0.291	0.301
F1610	3	2	2.000	0.693	0.058	0.503	0.500	0.332	0.375
F1625	3	2	1.964	0.684	0.068	0.494	0.491	0.448	0.370
F1672	6	3	2.045	0.880	0.330	0.514	0.511	0.309	0.456
F1703	3	2	1.938	0.677	0.071	0.488	0.484	0.757	0.367
F1760	10	4	2.367	1.019	0.215	0.581	0.578	0.352	0.504
F1908	6	3	2.993	1.097	0.852	0.670	0.666	0.074	0.592
F1940	3	2	1.337	0.419	0.023	0.253	0.252	0.313	0.220
F2068	3	2	1.266	0.366	0.080	0.211	0.210	0.556	0.188
F2074	3	2	1.872	0.659	0.080	0.469	0.466	0.379	0.357
F2185	3	2	1.225	0.330	0.000	0.185	0.184	0.227	0.167
F2202	3	2	1.933	0.676	0.023	0.486	0.483	0.375	0.366
F2281	3	2	1.458	0.494	0.016	0.317	0.314	0.786	0.265
F2288	3	2	1.996	0.692	0.114	0.502	0.499	0.273	0.374
F2290	3	2	2.000	0.693	0.138	0.503	0.500	0.240	0.375
F2305	6	3	2.896	1.081	0.193	0.659	0.655	0.203	0.581
F2370	6	3	1.815	0.683	0.636	0.452	0.449	0.179	0.358
F2382	3	2	2.000	0.693	0.011	0.503	0.500	0.425	0.375
F2540	6	3	1.293	0.463	0.091	0.228	0.227	0.187	0.214
F2551	6	3	1.372	0.532	0.136	0.273	0.271	0.145	0.254
F2734	3	2	1.839	0.649	0.159	0.459	0.456	0.328	0.352
F2782	3	2	1.294	0.388	0.057	0.229	0.227	0.335	0.201
F2901	3	2	1.600	0.562	0.023	0.377	0.375	0.218	0.305
F2979	3	2	1.146	0.249	0.023	0.128	0.127	0.262	0.119
F2019	6	3	1.406	0.561	0.034	0.290	0.289	0.198	0.269
BM114	6	3	2.377	0.936	0.897	0.583	0.579	0.043	0.487
BM136	3	2	1.133	0.234	0.011	0.118	0.117	0.345	0.110
BM212	6	3	2.848	1.071	0.818	0.653	0.649	0.150	0.574
BM289	3	2	1.727	0.612	0.102	0.423	0.421	0.346	0.332
BM295	6	3	2.630	1.028	0.609	0.623	0.620	0.189	0.546
BM306	3	2	1.576	0.552	0.000	0.369	0.366	0.745	0.299
BM341	3	2	1.789	0.633	0.094	0.448	0.441	0.810	0.344
BM344	3	2	1.839	0.649	0.000	0.459	0.456	0.203	0.352
BM374	6	3	1.630	0.707	0.188	0.389	0.386	0.675	0.354
BM378	3	2	1.969	0.685	0.511	0.495	0.492	0.140	0.371
BM396	10	4	3.636	1.331	0.796	0.729	0.725	0.173	0.673
BM411	3	2	1.999	0.693	0.277	0.503	0.500	0.313	0.375
BM483	10	4	3.258	1.248	0.309	0.699	0.693	0.315	0.633
F786	3	2	1.920	0.672	0.000	0.482	0.479	0.390	0.364
Mean	5.209	2.672	1.995	0.725	0.235	0.445	0.442	0.299	0.376
SD	2.766	0.786	0.722	0.302	0.300	0.173	0.172		

^a^ Number of genotypes where each locus amplified alleles; ^b^ observed number of alleles; ^c^ effective number of alleles; ^d^ Shannon's information index; ^e^ observed heterozygosity; ^f^ expected heterozygosity; ^g^ Nei’s (1973) gene diversity; ^h^ *F*-statistic value for evaluation of geographical differentiation; and ^I^ polymorphism information content.

**Table 2:** Distribution of allelic variation in 67 polymorphic simple sequence repeat (SSR) loci.

Number of Alleles	Number of SSR Loci	Polymorphic Loci (%)
2	34	50.75
3	22	32.84
4	10	14.92
5	1	1.49

**Table 3:** Estimates of genetic diversity within 11 populations of Chinese broomcorn millet.

Population	Genetic Parameter							
	N ^a^	Na ^b^	Ne ^c^	*I* ^d^	Ho ^e^	He ^f^	*H* ^g^	PIC ^h^
1	156	2.328 ± 0.877	1.843 ± 0.708	0.618 ± 0.377	0.239 ± 0.329	0.396 ± 0.230	0.380 ± 0.220	0.366
2	112	1.723 ± 0.781	1.650 ± 0.719	0.431 ± 0.421	0.215 ± 0.375	0.382 ± 0.358	0.287 ± 0.269	0.417
3	159	2.373 ± 0.775	1.821 ± 0.623	0.623 ± 0.336	0.197 ± 0.296	0.402 ± 0.230	0.309 ± 0.169	0.345
4	157	2.343 ± 0.897	1.892 ± 0.651	0.649 ± 0.367	0.217 ± 0.300	0.428 ± 0.225	0.404 ± 0.213	0.393
5	122	1.821 ± 0.815	1.552 ± 0.662	0.417 ± 0.395	0.230 ± 0.336	0.292 ± 0.273	0.264 ± 0.246	0.370
6	132	1.970 ± 0.797	1.653 ± 0.653	0.495 ± 0.375	0.206 ± 0.333	0.351 ± 0.255	0.313 ± 0.228	0.386
7	94	1.541 ± 0.673	1.472 ± 0.614	0.329 ± 0.382	0.271 ± 0.404	0.303 ± 0.345	0.223 ± 0.252	0.358
8	167	2.493 ± 0.823	1.934 ± 0.670	0.691 ± 0.322	0.263 ± 0.323	0.441 ± 0.188	0.427 ± 0.181	0.377
9	97	1.516 ± 0.713	1.483 ± 0.658	0.318 ± 0.405	0.188 ± 0.383	0.287 ± 0.354	0.215 ± 0.269	0.420
10	99	1.623 ± 0.662	1.515 ± 0.543	0.375 ± 0.376	0.240 ± 0.418	0.305 ± 0.299	0.254 ± 0.250	0.385
11	125	2.049 ± 0.845	1.709 ± 0.695	0.523 ± 0.380	0.331 ± 0.370	0.355 ± 0.251	0.332 ± 0.234	0.365
Mean	129.1	1.980 ± 0.361	1.684 ± 0.168	0.497 ± 0.133	0.236 ± 0.041	0.358 ± 0.056	0.310 ± 0.071	0.380

^a^ Total number of observed alleles; ^b^ number of observed alleles; ^c^ number of effective alleles; ^d^ Shannon’s information index; ^e^ observed heterozygosity; ^f^ expected heterozygosity; ^g^ Nei’s gene diversity; and ^h^ polymorphism information content.

**Table 4:** Number and distribution of alleles at each of 67 loci among 11 broomcorn millet populations.

Locus	Population										
	1	2	3	4	5	6	7	8	9	10	11
F265	2	3-D	4	3-D	3-D	4	2-AD	4	3-D	2-AD	2-AD
F258	3	3-C	3	3	3	3	2-B	3	2-BC	2	3
F334	2	1-B	2	2	2	2	1-A	2	1-A	1A	2
F503	4	2	3	4	3	3	2	4	3	1-A(h)	4
F510	2	2	2	2	2	2	1-A	2	-	1-A	1-A
F515	2	2	2	3	2	2	1-A	3	2	1-B	1-B
F619	2	1-A	2	2	1-A	2	2	2	2	2	2
F621	2	1-A	2	2	1-A	2	1-A	2	2	2	2
F630	2	1-A	2	2	1-A	2	1-A	2	2	2	2
F632	3	1-B(i)	3	3	2	3	2	3	2	2	3
F691	2	2	2	2	2	2	1-B	2	1-B	2	2
F1080	3	2	3	1-B	3	3	1-B	3	2	1-B	1-B
F653	3	2	2	4	4	2	2-CD	2	3	2-CD	2-CD
F1761	2	2	2	2	2	2	2	2(j)	1-B	2	1-B
F1036	2(a)	1-A	1-A	1-A	1-A	1-A	1-A	1-A	1-A	1-A	1-A
F1065	4	3	4	4	2	2	1-C(k)	4	2	2	4
F1429	4	4	4	4	2-AC	4	4	4	4	2-BD	4
F1400	2	1-C	3	3	2	2	2	3	1-B	2	2
F1387	4	4	4	4	3	4	3	4	2-BD	3	2-AD
F1380	5	3	4	5	3	4	2-CE	5	2-CD	3	4
F635	1-C	1-C	1-C	3	3	1-C	-	3	2	-	-
F746	3	1-B	2	2	1-B	1-B	1-B	3	1-B	1-B	1-B
F780	2	2	2	2	1-B	1-B	1-B	2	1-B	2	2
F836	4	1-B	3	1-B	1-B	1-B	-	4	1-B	-	-
F845	3	1-A	3	2	1-A	1-A	1-A	3	1-A	1-A	1-A
F850	3(b)	1-A	2	1-A	1-A	1-A	1-A	2	1-A	1-A	1-A
F1067	2(c)	2	2	1-B	1-B	2	1-B	2	1-B	1-B	1-B
F1071	1-B	2	2(e)	1-B	1-B	2	-	2	1-B	-	-
F1553	2	1-B	2	2	2	2	1-B	2	1-B	2	2
F1610	2	2	2	2	2	2	1-B	2	1-A	2	2
F1625	2	2	2	2	1-B	2	1-B	2	1-B	1-A	2
F1672	3	1-B	2	2	2	1-B	3	3	1-B	3	3
F1703	2	-	2	2	1-B	1-A	1-B	2	-	1-A	2
F1760	1-D	1-D	4	4	4	3	1-D	4	1-D	1-D	1-D
F1908	3	3	3	3	3	3	3	3	2-BC	3	2-AC
F1940	1-B	1-B	2	2	1-B	2	1-B	2	1-B	2	2
F2068	2	1-A	2	2	1-A	1-A	2	2	1-A	1-B	2
F2074	2	1-A	2	2	2	2	2	2	1-B	1-A	2
F2185	1-B	1-B	2	1-B	1-B	2	1-B	2	1-B	1-B	1-B
F2202	2	2	2	2	2	1-B	1-A	2	1-A	2	2
F2281	1-A	1-A	2	2	1-A	2	-	2	1-A	-	-
F2288	2	2	2	2	2	2	2	2	1-A	2	2
F2290	2	2	2	2	2	2	2	2	1-A	2	2
F2305	3	2	3	3	2	2	2	3	2	2-AC	3
F2370	2	1-B	2	2	2	2	2-BC	2(d)	2	2-BC	2-BC
F2382	2	1-A	2	2	1-B	2	1-B	2	2	1-A	2
F2540	1-A	2	2	2	1-A	1-A	1-A	2	1-A	1-A	2
F2551	2	2	2	3	1-A	1-A	1-A	3	1-A	1-A	3
F2734	2	2	2	2	2	2	1-A	2	1-A	1-A	2
F2782	1-B	2	2	2	2	2	1-B	2	1-B	1-B	2
F2901	2	1-B	2	2	2	2	2	2	2	1-B	1-B
F2979	2	1-B	2	1-B	1-B	1-B	1-B	1-B	1-B	1-B	1-B
F2019	2	1-B	2	2	1-B	2	1-B	2	1-B	1-B	1-B
BM114	3	2	3	3	2	2	2	3	2	2	3
BM136	2(f)	1-A	1-A	1-A	1-A	1-A	1-A	1-A	1-A	1-A	1-A
BM212	3(g)	2	3	3	3	2	2	3	2	3	3
BM289	2	1-A	2	2	2	2	1-A	2	1-A	1-A	1-A
BM295	3	3	3	3	3	3	2	3	1-A	2	3
BM306	1-A	-	1-A	2	2	1-A	1-B	2	-	1-A	2
BM341	2	1-B	2	2	1-A	1-A	-	2	1-B	-	-
BM344	2	2	2	2	1-A	1-A	2	2	2	2	2
BM374	2	2	3	3	1-B	2	-	3	1-B	-	-
BM378	2	2	2	2	2	2	2	2	2	2	2
BM396	4	3	4	4	3	3	2	4	3	2	2
BM411	2	2	2	2	2	2	2	2	1-A	1-A	2
BM483	3	2	3	3	2	2	1-C	2	1-B	3	4
F786	2	1-A	2	2	1-B	2	2	2	2	1-A	2
NOP ^a^	154	77	158	144	90	114	94	163	96	99	124
NOFP (%) ^b^	9 (13.4)	31 (46.3)	4 (6.0)	10 (14.9)	29 (43.3)	18 (26.9)	38 (56.7)	3 (4.5)	43 (64.2)	34 (50.7)	21 (31.3)
NOR ^c^	165		177					174			
NOUR (%) ^d^	6 (3.64)		2 (1.13)					3 (1.72)			

^a^ Number of alleles in the population; ^b^ number of fixed alleles (% of 67 loci); ^c^ total number of alleles in the ecotype; and ^d^ number of ecotype-unique alleles (% of alleles in the ecotype). Capital letters indicate fixed alleles, when two capital letters (AD or BC) appeared, it mean there have two fixed alleles in this population. Small letters in parenthesis mean ecotype-unique alleles. Parenthesis is used to distinguish with superscript small letters. A dash indicates that no alleles for the given locus were detected in the population.

**Table 5:** Distribution of broomcorn millet accessions from various provinces based on cluster analysis.

Province	Group A	Group B	Group C	Group D	Total Accessions
Heilongjiang	12	2	2	0	16
Shanxi	0	4	11	0	15
Inner Mongolia	11	16	5	0	34
Ningxia	0	3	7	1	11
Gansu	0	2	4	0	5
Shaanxi	2	2	2	0	5
Jilin	0	0	2	0	2
Accessions in each group	25	29	33	1	88

**Table 6:** Distribution of 88 broomcorn millet accessions based on STRUCTURE analysis (*K* = 3).

Province	Group 1	Group 2	Group 3	Total
Heilongjiang	1	13	1	15
Shanxi	6	0	9	15
Inner Mongolia	16	12	5	33
Ningxia	4	0	6	10
Gansu	2	0	4	6
Shaanxi	2	1	4	7
Jilin	0	0	2	2
Total	31	26	31	88

**Table 7:** Distribution of 88 broomcorn millet accessions based on STRUCTURE analysis (*K* = 11).

Province	Group 1	Group 2	Group 3	Group 4	Group 5	Group 6	Group 7	Group 8	Group 9	Group 10	Group 11	Total
Heilongjiang	7	0	1	0	0	0	0	6	0	0	1	15
Shanxi	0	0	5	0	1	1	5	0	0	0	3	15
Inner Mongolia	0	7	1	0	8	2	2	2	1	9	1	33
Ningxia	0	1	2	1	0	2	0	0	4	0	0	10
Gansu	0	2	0	0	0	0	0	0	4	0	0	6
Shaanxi	0	0	0	0	2	0	3	0	0	2	0	7
Jilin	0	0	0	0	0	0	1	0	0	0	1	2
Total	7	10	9	1	11	5	11	8	9	11	6	88

## Supplementary Materials

The supplementary materials are available online at www.mdpi.com/1422-0067/17/3/370/s1.
