# Supplementary material for: Genetic Diversity and Population Structure of Broomcorn Millet (Panicum miliaceum L.) Cultivars and Landraces in China Based on Microsatellite Markers
Source: Int J Mol Sci. 2016 Mar 14;17(3):370. doi: 10.3390/ijms17030370 (PMC4813230; doi:10.3390/ijms17030370)
Supplement: Supplementary file 1 [file ijms-17-00370-s001.pdf]

Supplementary Materials: Genetic Diversity and Population Structure of Broomcorn Millet (*Panicum miliaceum* L.) Cultivars and Landraces in China Based on Microsatellite Markers

Minxuan Liu, Yue Xu, Jihong He, Shuang Zhang, Yinyue Wang and Ping Lu

Table S1. Broomcorn millet varieties and landraces used in this study.

No.	Population	Ecotype	Name	Pedigree
1	1	Northeast	Longshu21	Longshu5/Longshu9//Longshu10
2	1	Northeast	Longshu5	
3	1	Northeast	Longshu10	
4	1	Northeast	Longshu9	
5	1	Northeast	Longshu23	Longshu21/Longshu3//Longshu16////Longshu2//Xiaonangouheimizi///Longshu5
6	1	Northeast	Xiaonangou Heimizi	
7	1	Northeast	LLongshu12	
8	1	Northeast	Longshu3	
9	1	Northeast	Longshu16	
10	1	Northeast	Longshu2	
11	1	Northeast	Nianfeng5	
12	1	Northeast	Nianfeng2	Nianfeng2/unknown
13	1	Northeast	Niangfeng2r	
14	1	Northeast	Qishu1	62green1/unknown
15	1	Northeast	Nianfeng7	Unknown
16	2	Northeast	Jiushu1	Huangmizijiu58 system selection
17	2	Northeast	Panlong Huangmizi	Unknown
18	3	Alpine Region	Yanshu7	Yixuanhuangshuzi/Dabaishu
19	3	Alpine Region	Jinshu6	Unknown
20	3	Alpine Region	Yanshu8	Unknown
21	3	Alpine Region	Jinshu8	Erbaishu/8106-981-1//34-22
22	3	Alpine Region	Jinshu4	Neishu2/yishu1
23	3	Alpine Region	Jinshu5	981/Yishu1
24	3	Loess Plateau	Jinshu7	Neimenghongshu/Shanxixiaohongshu
25	3	Loess Plateau	Pinmi1	Huangmizi system selection by mutation
26	3	Loess Plateau	Jinshu1	8114-15-8/8106-983-3
27	3	Loess Plateau	Jinshu3	Ziluodai system selection
28	3	Loess Plateau	Ziluodai	
29	3	Loess Plateau	Jinshu2	Tianzhenshuzi system selection
30	3	Loess Plateau	Tianzhen Shuzi	
31	3	Loess Plateau	Jinshu9	8114-15-8/unknown
32	3	Loess Plateau	8114-15-8	
33	4	Northwest	Ningmi10	Haiyuanziganhong/unknown
34	4	Northwest	Haiyuan Ziganhong	
35	4	Northwest	Ningmi15	Ziganhong/unknown
36	4	Northwest	Ziganhong	
37	4	Northwest	Ningmi5	Unknown
38	4	Northwest	Ningmi11	Yu 6—14 system selection
39	4	Northwest	Ningmi13	70-1046/ZiganDariyue
40	4	Northwest	Ningmi14	Gugutou/62-02
41	4	Northwest	Ningmi16	Guyun5 system selection
42	4	Northwest	Ningmi17	Gugutou/62-02
43	5	Northwest	Longmi3	Ganmi1/unknown
44	5	Northwest	Ganmi1	
45	5	Northwest	Longmi4	XiyanbeiDahuangshu/Huiningdahuangmi
46	5	Northwest	Longmi5	Yemizi/Benlanjidanqing//Fengshou-4

Table S1. *Cont.*

No.	Population	Ecotype	Name	Pedigree
47	5	Northwest	Longmi7	8116-2-3-1/8428-1-1-2
48	5	Northwest	Longmi8	7814-11-1-2-3/Fu7705-4-4-2
49	6	Loess Plateau	Niuluandanmi	
50	6	Loess Plateau	Yumi2	Shenmuhongmizi system selection
51	6	Loess Plateau	Shenmu Hongmizi	
52	6	Loess Plateau	Yushu3	Unknown
53	6	Loess Plateau	Yushu2	Unknown
54	6	Loess Plateau	Yushu1	Unknown
55	7	Loess Plateau	Zigang Hongshu	Landrace
56	7	Loess Plateau	Zigang Hongshur	Landrace
57	8	Mongolian Plateau	Yixuan Huangmi	Zhunqihuangshuzi/unknown
58	8	Mongolian Plateau	Zhunqi Huangshuzi	
59	8	Mongolian Plateau	Neimi5	Neimi3/Yixuandahongmi
60	8	Mongolian Plateau	Neimi3	
61	8	Mongolian Plateau	Yixuan Dahongmi	
62	8	Mongolian Plateau	Chimi2	Neimi5/unknown
63	8	Mongolian Plateau	Yimi5	Niuluandanmi/unknown
64	8	Mongolian Plateau	Neimi7	Linhehuangmi/Zhunqihuangshuzi
65	8	Mongolian Plateau	Linhe Huangmi	
66	8	Mongolian Plateau	Neimi6	Daqiqingmizi/Helindahuangmi
67	8	Mongolian Plateau	Daqi Qingmizi	
68	8	Mongolian Plateau	Helin Dahuangmi	
69	8	Mongolian Plateau	Heling Dahuangmir	
70	8	Mongolian Plateau	Neimi3	Dangdidahuangmizi system selection
71	8	Mongolian Plateau	Dangdi Dahuangmizi	
72	8	Northeast	Chishu1	Dahongshu system selection
73	8	Northeast	Chishu2	Dangdidabaishu/unknown
74	8	Northeast	Chimi1	Huangmizi system selection
75	8	Mongolian Plateau	Neimi1	Unknown
76	9	Mongolian Plateau	Xiaobaishu	Landrace
77	9	Mongolian Plateau	Xiaobaishur	Landrace
78	10	Mongolian Plateau	Dongsheng Erhuangmi	Landrace
79	10	Mongolian Plateau	Dongsheng Erhuangmir1	Landrace
80	10	Mongolian Plateau	Dongshegn Erhuangmir2	Landrace
81	11	Mongolian Plateau	Ziganhongmi	Landrace
82	11	Mongolian Plateau	Ziganhongmir1	Landrace
83	11	Mongolian Plateau	Ziganhongmir2	Landrace
84	11	Mongolian Plateau	Ziganhongmir3	Landrace
85	11	Mongolian Plateau	Ziganhongmir4	Landrace
86	11	Mongolian Plateau	Ziganhongmir5	Landrace
87	11	Mongolian Plateau	Ziganhongmir6	Landrace
88	11	Mongolian Plateau	Ziganhongmir7	Landrace

1–10 were provided by Institute of Crop Science, Heilongjiang Academy of Agricultural Science; 11–15 were provided by Nenjiang Institute of Agricultural Science, Heilongjiang Academy of Agricultural Science, China; 16–17 were provided by Jilin Academy of Agricultural Science, China; 18–23 were provided by Institute of Gaohan Crop Science, Shanxi Academy of Agricultural Science, China; 24–32 were provided by Institute of Genetic Resources, Shanxi Academy of Agricultural Science; 33–42 were provided by Guyuan Institute of Agricultural Science, Ningxia, China; 43–48 were provided by Gansu Academy of Agricultural Science;4 9–56 were provided by Yulin Institute of Agricultural Science,Shannxi, China; 57–88 were provided by Inner Mongolia Academy of Agricultural Science, China.

Table S2. The information of SSR primers used in this study.

Primer Name	Forward Primer 1 (5′–3′) Seq	Size	Reverse Primer 1 (5′–3′) Seq	Size	Length
F255	CGGTAGAGAGAGCCAGCG	18	CTCTTCCTCCTCCAGCTCCT	20	203
F256	GGGAACATGTTTAGGGGCTT	20	TGGTTAATAGCCCCACAAGG	20	225
F257	CTCAAGGTGTGGGTGTGTGT	20	TCGGAAATAAAATAGGTTTGTACC	24	163
F258	GTGTCTCTTTCGTCTTGCCC	20	GGGACACTTCCACCATCATC	20	204
F259	GTGCTTGACAAATTCCGGTT	20	CCACGCCTCCTGTAACTACG	20	293
F264	GAGAAAGAGAGCGAGCGAGA	20	CGAAAATAACCAACCGACCA	20	220
F265	GGCTTTGCTAGGGTTTCTCC	20	GGTGTGAAGTTGCCCAGATT	20	226
F266	GCTTCTTTTTATCCGAGGGG	20	AGGGTATCCGGTGCTTAGGT	20	216
F267	GGGTTACCCGGATGGTAGAT	20	TCACTCGAGAATCATCACGC	20	283
F268	GCGATCGTAGTCGATAAGCC	20	TCCTTGCTTGCACATCACAT	20	287
F269	CGGAATGGGAGGAAGAGAAG	20	ACCCTCCCTACCACCTCCTA	20	181
F270	TGCAACCCTCTTCCCTATTG	20	TCCTCCACATCAGAACCACC	20	187
F301	AGGTCGGTGTCTGTACCCTG	20	ACTAGGTTGACCATGACGCC	20	289
F302	AATCAGTGCCTTGCTGCTTT	20	GTAGGCAAGGCAGTTTCAGC	20	228
F303	AGGAATTGGATTCCCGAAAG	20	TTCTCTCTCTCACTCTCTCCCC	22	249
F304	GGGAAGAAGAAACACAAGCG	20	ATCCAGGGAGCTCGAATCTT	20	187
F305	CCTTCCCTTGCTTCTCTCCT	20	ACACACACACACACACACGG	20	235
F306	ACATGTTCATGCATGTGGCT	20	GCAGTCTGGTCCAAGAAAGG	20	230
F307	ATCACTGCTGTTTGGGGTTC	20	GGTAGTGAGGAGTGGCGGTA	20	284
F308	ATGCATGGATAGAGGGCTTG	20	TCCGTCCACTCTTCGTCTCT	20	232
F309	AGACGAACCGGGTCAGGTA	19	AAAGCCTCTGCCTTCTCTCC	20	207
F310	ATAGAGCACCTGCTGAGCGT	20	CCTTACATTTTTCCTTTGTCCA	22	274
F311	TAGCCGCAGATTATCAGCCT	20	ACACACACCACACACCACG	19	294
F312	AGGTGTGGTTTCTGGTCTGG	20	TAGGGATCGTCCAATGCAAG	20	296
F313	CAGGCCATGATTCCTTTGTT	20	ACGGTTCCTATTCCTCACGA	20	283
F314	GCCTCAAGATCGTCAAGCTC	20	GTGTGCAAACAAAGCCGAT	19	260
F315	GCCTCAAGATCGTCAAGCTC	20	GTGTGCAACAAAGCCGATCT	20	259

Table S2. *Cont.*

Primer Name	Forward Primer 1 (5′–3′) Seq	Size	Reverse Primer 1 (5′–3′) Seq	Size	Length
F316	TCTTTTATGCGCGTAAGGCT	20	TCACTTGTAACCCCGGTAGC	20	297
F317	CAGTGTTGGCTAAGGTGGGT	20	GTAGCGCACGACATAACTGC	20	256
F318	ACAGTCCTGACGGCAAATTC	20	CTCAAAGCGGTAAGGTCAGC	20	261
F319	TGCGGCTGGCTAGAATACTT	20	ACGCGACGAACGAACAAC	18	230
F320	AAGAGATGGTGCGCTTGAGT	20	ACCTGTAAGGCTGTCCCCTT	20	237
F321	AGCAGCCTAGGCCTCTTCTT	20	CACACAACAACACACATACACACA	24	292
F322	CGGCCAGTGAGGAGTTACAT	20	TCTTGATGGCATGACCTTTG	20	280
F323	TGACTAGCACTTGGGAAGGG	20	GATTTTCAACACATGCACGG	20	223
F324	GGTCGCTCGTAATGTTTCGT	20	GCAGGAGAGCTGCGTATCAT	20	237
F325	CGCGCTAAGTGTTGAGTGAG	20	ACCTGTAAGGCTGTCCCCTT	20	173
F326	CCAGTCCTAGCTAAGGTGCG	20	AATTCCCTACTCGGACTCCC	20	295
F327	TCCAGCTAGGGTCAGTCGAT	20	TTCTAAATAGACACAAGAGACGTGC	25	183
F328	GCCGTAGGAGAGGTTGAGAG	20	GCCTGTTTTTGTTAGGCTGC	20	273
F329	AATGCCAGAGGAGTGGGTTA	20	GAAACTTGGAAGGTGCTCCA	20	210
F330	GAGCATCCTTCTTGGAGCTG	20	GGCCAAAGCAAATGTCAAGT	20	237
F331	GTATGAGGTGTTGCTCGGGT	20	TCTCACTTGTAACCCCGGTC	20	227
F332	GGTCGCTCGTAATGTTTCGT	20	GCAGGAGAGCTGCGTATCAT	20	243
F333	TGGGTTAGGAGGTCAAGAGG	20	TGCTCCAGTGTGCAACATTT	20	186
F334	TGTCTAGACCATCGCCATCA	20	CACTCACACACACATTTTCTTGG	23	218
F335	ATTGGACGAGTTACGGCTTG	20	CATTGAGTGGGGTTGATCCT	20	191
F336	TCTTGTTCTGTACAGTTTGTATGGG	25	ACCATTGGCCGATATTACCA	20	212
F337	AATGCCAGAGGAGTGGGTTA	20	GAAACTTGGAAGGTGCTCCA	20	213
F338	TACGCGGGGTCTGTAGTAGG	20	TCTCACTTGTAACCCCGGTC	20	203
F339	AGAGAGCGAGACAGACAGGC	20	AGTGTCAGAAGGTGGGGTTG	20	213
F340	CCAGTTGGGAAAACTCTCCA	20	TTGTCGTTCTCACTATGCACAC	22	180
F501	CACCAATCAACAACTCGTGG	20	TCGGAGTGGAACGAAGTACC	20	239
F502	ACTTGGGTCCCTCCTCTTGT	20	GTCTCTTGGGTGTGCACTGA	20	217
F503	GTGGTACAGCTGCTCGTTCA	20	AGGAGGAACCAGGAAGCAAT	20	254

Table S2. *Cont.*

Primer Name	Forward Primer 1 (5′–3′) Seq	Size	Reverse Primer 1 (5′–3′) Seq	Size	Length
F504	GCAAAGCCCACCAAATACAT	20	GTTGTGTGGTGCCGTGATAG	20	253
F505	TGGCACACGTAACACGGTAT	20	TCTGGACCTGCGAGCTTAAT	20	154
F506	TTAACCGAACAAGGAGTGGG	20	CTAAGCACTTGGGCGATG	18	167
F507	AGCAGCAGAACATGCACATC	20	TGTCATTTTCATTTTAATTGGTAAAGT	27	162
F508	AGCGAATTTTCTTGCAGTGG	20	CGAGTTCTTCTCAGGGTTGC	20	269
F509	TTCTTACGGAAGTTGGGGTG	20	TTTCTAATTTGGTCATTCGCAA	22	258
F510	GTGGTACAGCTGCTCGTTCA	20	CAAGACAGGGAAGGAACCAA	20	268
F511	ATGGCACTTTTCAACGCACT	20	TTTGTTTTGTTGTCGGCGT	19	167
F512	TTCTTACGGAAGTTGGGGTG	20	TCAATTTGGTTCATTCGCAA	20	261
F513	AAGGCTTAGGCCCATCAAGT	20	CAAACGAGTTTGTTCTGCTGTT	22	211
F514	TGGCACACGTAACACGGTAT	20	TCTGGACCTGCGAGCTTAAT	20	159
F515	CGTTTTCTCGCTACACACGA	20	TGGACAACGGAAAACGTACA	20	194
F516	TCAGCAAGATGCAAAACGTC	20	GATGCAGCTGGTGCTACTACT	21	150
F517	GCCACGCCACAGTTTTTAAT	20	TTTGTTATTCTATGCATGTATTCGTT	26	202
F518	TTAACCGAACAAGGAGTGGG	20	GACGAGTTACGGCTGGTCAC	20	193
F519	CCCTCTCACACACACACGAG	20	TAGTCCGAACGTGGACCAAT	20	248
F520	TCTTCTTACGGAAGTTGGGG	20	CAATTTGGGTTATGTTCTTCTCG	23	167
F611	CAGGGAGTGGTGTTGATCCT	20	ATTGGACGAGTTACGGCTTG	20	180
F612	GCTTCCAACTTCAACTTGCC	20	GCTAGGCATGTGCTCACGTA	20	264
F613	AGGCGGACCGAATTTTTACT	20	CCATAACCAAGACATGAGCG	20	207
F614	CATTGAGTGGGGTTGATCCT	20	AATGGACGAGTTACGGCTTG	20	181
F615	TCCATTCATCAGACCACAGC	20	TGGGGAGTTGGGAGTAGTTG	20	177
F616	GCTCACAAAGATGACCCCAT	20	TTTGTTTTCCCTCGCTCTTC	20	212
F617	AATGCAAACAACAGAAGGGG	20	GGAGTGACCTCTCTCCCTCTC	21	192
F618	AGGCGGACCGAATTTTTACT	20	GTGGCCGACGTCTGTAAGG	19	171
F619	CTGCTGCATGCCTTTACCTT	20	CGCTGCAGTTTTGGTCAGTA	20	252
F620	CATTGAGTGGGGTTGATCCT	20	GCATTGGACGAATTATGGCT	20	180
F621	ATGAATCACCCGATCCACAT	20	ACGCCAACATCAGCATATCA	20	209

Table S2. *Cont.*

Primer Name	Forward Primer 1 (5′–3′) Seq	Size	Reverse Primer 1 (5′–3′) Seq	Size	Length
F622	CACTGTATTATCCTCCCCCG	20	CCTGGCCTTGTTCTCATCTC	20	189
F623	TGAAAAACGAAGGGGATCTG	20	ATTGGACTTACAACGGGGTG	20	265
F624	AGGTTTTTCAAACCACGCAC	20	TGTCTTGCTTCGTTTATGCG	20	203
F625	CACTGAGTGGGGTTGATCCT	20	ACGAATTACGGCTTGGTCAC	20	179
F626	GTGGATGGCTGTGCCTTTAT	20	TGTTTGTCCTAACGGAAAATAGG	23	188
F627	TATCCACCAACGTGGGATTT	20	CTACGGAAGAGAGTGGCAGG	20	219
F628	GTGTAGCCAGGATCAGCCAT	20	GCGGTAGATGGATTGAACGA	20	181
F629	AGGTTTTTCAAACCACGCAC	20	CCGACTGTCTGTCTCCGTTC	20	183
F630	ATGAATCACCCGATCCACAT	20	ACGCCAACATCAGCATATCA	20	211
F631	GTTTGGTCTCCTGCAAGCTC	20	CGTGTCTTCGTTATGTGTCCC	21	279
F632	GCTGTCGGTCAGTCCTGTTT	20	ACGCCAACATCAGCATATCA	20	161
F633	ACCCCATCCTTTCTCCAAAT	20	TTCGATGGCTAGCACACAAG	20	278
F634	TTTATTATCGCCGTCGTTCC	20	CCTTCCTGCTGGCGTAGTAG	20	187
F635	GCACACGCATCATCACAAGT	20	GCTCATTCAACGACAGATGC	20	280
F636	CCAAGCTTTATCGCTATCGG	20	TGTGTGTGTGAGTGTCTGTGTG	22	196
F637	ATACCCGAAGCATTGAGTGG	20	GTTTGGGTCGAAGAATGTCA	20	169
F638	GCACACGCATCATCACAAGT	20	CAACGACAAGATGCAAGACTG	21	275
F639	TTAACCGAACAAGGAGTGGG	20	ACATAGCTAGCACTTGGGCG	20	179
F640	CTGGTACTCCCACAAGCGTT	20	GTTTTCCTTTCGTCTGCTGC	20	237
F651	CGAAGCATGTTACTCCCCAT	20	GACGGACGAGAGCATGTGT	19	204
F652	CGAAGCATGTTACTCCCCAT	20	TGTGTGCGTACATATAATACGGTG	24	190
F653	CGATGAACGAAAATTCACCC	20	GTTCATTCGTCCAAATGCCT	20	258
F654	CGTTTTCGAAAGGGTTTTTG	20	TGTTTGGGTCGAAGGAGTGT	20	216
F655	GCCACTTCCAATCACAATCC	20	GGTTCGTATTCGTCCAACTAGC	22	172
F656	CATTGAGTGGGGTTGATCCT	20	ATTGGACGAGTTACGGCTTG	20	176
F657	AAGACGGCAGACATACAGGG	20	CTCCGTACATTTTGTGGCCT	20	170
F658	CAATGAGTGGGGTTGATCCT	20	GCATTGGATGAGTTACGGCT	20	183
F659	CATAGCTCACGGCACAACTG	20	CGTGTTGCGGTACCCTACTT	20	197

Table S2. *Cont.*

Primer Name	Forward Primer 1 (5′–3′) Seq	Size	Reverse Primer 1 (5′–3′) Seq	Size	Length
F660	TCGAACATGCAGGAAAACTG	20	TTTCCCTTTCAGGCAATCAG	20	164
F691	ACTCATGGTTACGGCAACTG	20	GCGCGAGAGAGAGAGAGAGA	20	287
F692	CTCTGTGTTTGTTTGGGGGT	20	GCAAGGACCTCTAATGCACC	20	235
F693	CCGACACTACTCTGCTTCCC	20	GACAGCGGTTACCTTCCGTA	20	166
F694	CCTCCCGTCCTAGTGTACCA	20	GTCACATTGTCACTGCGTCC	20	262
F695	GGCAATCGATGTAAGGTCAGA	21	TCTTTGCAAATTTTCCGGTC	20	207
F696	AATTCAAAATTCCAGCACGG	20	AGTAGGAGGAGGAGGGAGGG	20	189
F697	AGGACAGGCCATTTATGTTTG	21	ACCTCCTACTGCGTTCGCTA	20	218
F698	GAGGTGAGAGACTTGACGGC	20	GATCTACGGCCAGGAACACT	20	181
F699	AGTGCCCAATGACCAAAGAC	20	GCGACTGGTACGTCCTTTTT	20	176
F700	CTGCTCTTTGGCTCTCTGCT	20	GGTGGTAAGGTAGGTCCCGT	20	176
F741	GGACAGCAGCATACAGGACA	20	GTACGTCGACTTCCTACCGC	20	185
F742	AGTACCAACCCAGCATCAGG	20	AAGGACAGGTCCATTTATGTTTG	23	267
F743	CCAGTTTGGGAGCAACAAGT	20	GTTGAAGCTCAGGGAGAGGA	20	222
F744	AATAAATTGAAATCCACCCGC	21	CTCTACTCTCTCCCTCCCTCC	21	223
F745	CGAATGTAGGTTGGGACTGG	20	GAGCTCGTCCTCCAACGAC	19	254
F746	CGTCCACCTTGGTGCTTATT	20	GCTGATTTTCTAACGGCTGC	20	236
F747	TGAAAGGATGGGGAAACAAC	20	CATTATACTCTCCGCTGCGT	20	195
F748	CAAGCCCATACAATGCCTTT	20	ACAAGATGGACAGGTGGAGG	20	168
F749	CAGTTGCCTCACCTTGCTC	19	CCGCTCCTTACTCCTCTCCT	20	206
F750	AGTACCAACCCAGCATCAGG	20	TTTCTACTAATTCCTTTCGTTGTTCA	26	247
F771	ACTGATGTGATCTAGCCCCG	20	CCACCCAACGGAGTCATC	18	173
F772	GAGAGATCAGTGAGGACGGC	20	ACCCCTCCGTACGCTACTAC	20	228
F773	AGCACACACGACGAGTTCAC	20	ACCACAACCAACCACACACA	20	181
F774	GTTGCACCACACCAACACAT	20	GTCCTCGCACGAAGACTACC	20	164
F775	TAGCCCGTAGTGCTCCAACT	20	CGACGGTTTCGTACTCTTCC	20	223
F776	GCGAGCTATACCTCTCACCG	20	TGTGTAAGAGAGAGAAGAGTATGTGTG	27	160
F777	ACTCAGCGATTGTGTTGCTG	20	ACGGAGTCATCATGTCGCTT	20	172
F778	TGACTGCAAAAAGGATGCTG	20	CCAACCACCACTACTACGCC	20	201

Table S2. *Cont.*

Primer Name	Forward Primer 1 (5′–3′) Seq	Size	Reverse Primer 1 (5′–3′) Seq	Size	Length
F779	TGGAGCAGCAACAAAACAAG	20	TCGGTGGTACGGACTAGTGT	20	191
F780	CTGCATTCTCTGTTCACCCA	20	ATCCTTTCACTCGAGGGGTT	20	250
F781	CGGCGAAAAAGCTCACTAAT	20	CTTCCTTCCTTCCTTCCTCC	20	246
F782	GTATCAAGCCGGAGAATGGA	20	CCGTTACGTTCGTTTCGTTT	20	235
F783	ACGTCGATGGTGCATAAACA	20	TCGTCTCTCTACGTCTACTCTCTCT	25	177
F784	GCACTGGACCAGGTTTTCAT	20	TCTCTCTCTACTCTACTCTCTCTCGTC	27	204
F785	GCCATGTTGCTTGTTGACTG	20	TCGTCCTACTCCTACCTCTCCTC	23	219
F786	CCTGGACACACACACACACA	20	TCTTGTCACTGTCGGCGTAG	20	233
F787	TACCATTGTTAGGGCCTTGC	20	CCTACCGATCTAGGTTTGCG	20	276
F788	ACAAAGAAGCGATGCGAGAT	20	AATATCCGGCTTGACACAGG	20	172
F789	GGTGGGTGACAGTGCGTAG	19	CTTGGAGGCCCAAACCTAGT	20	239
F790	AGATTGGATTTGAAGGGGCT	20	TTCACCCAAAGATCATCACG	20	271
F831	GTTACAGGAGCGCGAGAGAG	20	TATAAAGGAAGAACGGGGGC	20	154
F832	AGCTGTGGAAGAACGGAAGA	20	CAGAACTAGCGCAAACAATGA	21	210
F833	AGGCAAGTCATCCTTGGCTA	20	CGTTCTCCGTTGAGAGGAAG	20	238
F834	AGCCACCGCTAGAATGCTTA	20	CCCACACAAGGAATACCCAT	20	193
F835	CACGAGCCCAGAAGTCCTAC	20	TTGGTACCGCAATACGTGTC	20	275
F836	GCGCAGTAATATATTTCAGTAATTCA	26	GCATCATCGTCAAGACCTCA	20	225
F837	GCAAGCAGGGTAGGAATGAC	20	CTACGCTCCCTCCAAGACAG	20	159
F838	AGCTGTGGAAGAACGGAAGA	20	ATCTGTTCATTGGGAAACGC	20	249
F839	CGGACTAGCCAAGGAGACTG	20	GGAACGCACTGCATAGCATA	20	242
F840	GCCATTGCCACTGATCTCTT	20	CGTTCTCCGTTGAGAGGAAG	20	202
F841	ATTGGACGAGTTACGGCTTG	20	TTATTCGCTATCGGACTCCC	20	155
F842	CTCGTCTCCTCCCTCCCTCT	20	CCAACCTCACCACTCCACTC	20	168
F843	CGTAGGACGACTGACTGTGC	20	CGACCCACGATAAGGAAGTC	20	156
F844	CCTATGTCCAGTCGGGGTTA	20	ACCACGAACACAAGAGAACG	20	233
F845	GCGCAGTAATATATTTCAGTAATTCA	26	GCATCATCGTCAAGACCTCA	20	226
F846	TAGTTGATGCGTCTCGTGCT	20	GGCAGATCCCCTACTCATCA	20	223
F847	TCTTTTATGCGCGTAAGGCT	20	CGAGATAGGGACGTCAAAAAG	21	262

Table S2. *Cont.*

Primer Name	Forward Primer 1 (5′–3′) Seq	Size	Reverse Primer 1 (5′–3′) Seq	Size	Length
F848	AGCTGTGGAAGAACGGAAGA	20	TCAGAACTAGCGCAACAATGA	21	216
F849	CCTATGTCCAGTCGGGGTTA	20	CACGAACACAAGAGAAGTAGCTC	23	232
F850	GCGCAGTAATATATTTCAGTAATTCA	26	GCATCATCGTCAAGACCTCA	20	227
F1031	CGTTCTTTCTTTTGTTTGTTACG	23	CGCAGCAGCAGGGAGTAT	18	154
F1032	GGCCCCTGAGATAGAGGACT	20	CGGGGTCGTATAGTAGGGGT	20	156
F1033	AACGCAACACGAACACACAT	20	TCTTCCTTCTACCCGTCGC	19	216
F1034	AAACACAAGCCGAATTGTCC	20	ATCGCCTGTCAATTTCTTGG	20	214
F1035	CTGAAACGTTCAGTGGGGTT	20	AGCACTTGGGTAAGTAGGAGTG	22	174
F1036	CAGAGCAGTGCGGTATTGTG	20	TCGTTTGTTGTTCGGTTGTC	20	232
F1037	GAGACGCCTTTCTCAACCAG	20	TTCGATTTGTCTCGCTTCCT	20	196
F1038	ATTCAGCGGCATAATCCATC	20	CTTCTCGCACAAGACCACAA	20	261
F1039	AAACACAAGCCGAATTGTCC	20	GTGTCTCTTGCCCTCGCTT	19	187
F1040	TTAACCGAACAAGGAGTGGG	20	TACGGTCGTTGGTTCGTACA	20	196
F1061	GGGAACACGAACACATGGTA	20	CAGTGATGATCCTGGATGGA	20	264
F1062	GCCACGCCACAGTTTTTAAT	20	CGTGTGTATATTTTGTTTATTCTATGC	27	218
F1063	GCATCGTTTTCCTCTTTCCA	20	CTCCTCGTCTCTCCCTCCTC	20	257
F1064	CTGTCGTTGCTTCCCTTCTC	20	TCCCTTTCCCTTTTCCTTTT	20	240
F1065	TCTGGACATGCTTTCACCAG	20	CCTACCTCGTAACACTGCGG	20	267
F1066	GCATCGTTTTCCTCTTTCCA	20	GTCTCCTCCCTCCCTCCTC	19	255
F1067	ATCGACGACTAGGCCCTGTA	20	TGCGGAGTGTCTTTGTTCTG	20	199
F1068	CTGTCGTTGCTTCCCTTCTC	20	ACGGTTAGTCGACGCGAG	18	180
F1069	GGGCATGTGCTTTTTCTCAT	20	ATAGGTAGGAGGCGATGTGC	20	181
F1070	GTGTTGATCTGCACCCACC	19	CGAGCATTTCATGGAGATCA	20	182
F1071	ATCGACGACTAGGCCCTGTA	20	TGTATGGAAAGCTCTGGCCT	20	159
F1072	TCTGGACATGCTTTCACCAG	20	TACCTGTAACACTGCGGCTG	20	266
F1073	ATGATGTGATCTGGCTGACG	20	CGCTTCAATACGTTGTCCTG	20	236
F1074	TCTGGACATGCTTTCACCAG	20	CACTGCCTGATCCTACCCTC	20	286
F1075	CCATCACAATGCGAACTTCA	20	GGGAGTCTATCCTCGACATACG	22	182
F1076	TACGTCAACGATACCTGCCA	20	GGCGACAGTAGGTGCTCAAT	20	182

Table S2. *Cont.*

Primer Name	Forward Primer 1 (5′–3′) Seq	Size	Reverse Primer 1 (5′–3′) Seq	Size	Length
F1077	CCAGCCATTGTCCTATTCTGA	21	GTGTGCTCTGGCCTTTTCTC	20	217
F1078	TACATCGATGCCAATGGAGA	20	TAGAGGGAGGTTGGTCGATG	20	175
F1079	CGAGACGCTAAAAGGTGAGG	20	TTCTTCCTTCCCTACCTAACTTAAC	25	261
F1080	ATCGACGACTAGGCCCTGTA	20	GGCCGTCACTATATCTGTCACC	22	153
F1371	CACCCACCAAACAAACACAG	20	CCTAGATCTCGCTCTGGAGG	20	250
F1372	TGGAGTAGCAGAGAGGGAGC	20	GGAAAAGAGGTTTGGGGAAG	20	264
F1373	TGTAGCTGCACCGGAATATG	20	TCTCTCTCTCTCTCGCCCTG	20	291
F1374	CTCTGACCGCATCTGAAACA	20	TTAGACAGCCAGCCACTTCA	20	297
F1375	CGTTGGTTGCTTGACAAATG	20	TGTAGGTGCTGGAGCTTGTG	20	201
F1376	TTACTTGTAGGCGACCGACC	20	ATGATGGGGTGTACGCAGTT	20	172
F1377	TCAGCACATTTCTGACCAGC	20	TCCGACCCTACGTCGTAACT	20	140
F1378	TTTGACTCATCATCTCAAGTATCCA	25	TGTGTGTGAGTGTGTATGTGTGTG	24	119
F1379	TTTGACTCATCATCTCAAGTATCCA	25	TGTGTGTGAGTGTGTATGTGTGTG	24	120
F1380	GCCTCCTGTCTTGTAGCGTC	20	AGGGTAGGCTGAGAGCCTGT	20	121
F1381	TTCTCTCTCTCCCAGTCTCCC	21	AACGGTGTTCCCAAAGACAC	20	147
F1382	TCCATGTAAAGGCTCTCGCT	20	CTGAGGTCCTTTGACGAAGC	20	155
F1383	AAAGGAACAGAAGCAAAGCG	20	TTGCCTTTTGGAAGCTGTCT	20	116
F1384	TGGAAACTCCATGGACAACA	20	AGTCTGACTCGGTCGTCTCC	20	252
F1385	TCAACGTTTCTAGCTTGCCA	20	GTGCATTGTTCATGATTGGG	20	141
F1386	ATATCAAATTGCGGCACACA	20	CCGTACGTCTCTCGTTCTTTG	21	130
F1387	TTTCAGGGACTGGACTGGAC	20	GTAGGGGGTAGCTGAGAGCC	20	105
F1388	TGGGTGCGAGTACTACCAGTT	21	AAGACCTGCAGGAGCTTCAC	20	101
F1389	TTTTATCAGAGGCAATCCCG	20	ACCAGTGCATCGAATGTTCA	20	287
F1390	GCAGTCGTGCCTCTCCTTTA	20	GGGAGAGAGAAGTGAGATGGG	21	118
F1391	CAGAAGGCTTTGGGAGACTG	20	CCTCCCTCCTGTGTTACGTC	20	206
F1392	CTGGACCCACAATGAGCTTT	20	ACTCAGTAATCACGCGTCCC	20	182
F1393	ACCCCTTGAACTGCACAAAC	20	CGCTGCCATGTAGAGACAGA	20	151
F1394	TATCCCCCTTTCCAAGCTCT	20	GCACAGCACAGCACAAAACT	20	193
F1395	GAGCCACCATCAACCCTTTA	20	AAATCTCCGGTGAGAAATCG	20	240

Table S2. *Cont.*

Primer Name	Forward Primer 1 (5′–3′) Seq	Size	Reverse Primer 1 (5′–3′) Seq	Size	Length
F1396	GTGGGATGTGTAGCCGAGTT	20	GGAGCAGGTGTGGATACGTT	20	288
F1397	GATCCACCTCTCTCTTCCCC	20	AGAGAGACCCAAGACCTCGC	20	152
F1398	GTCACGGCGTGTGTGTGTAT	20	GTGTGGGTGTGGTTCTTGC	19	300
F1399	TCTCTAGGGTGGATGGATGG	20	TAGGTGAGGGGTGGTACTGG	20	150
F1400	AGCAACGGAGGTGAGAGAGA	20	TCGACACACACGACACACAC	20	128
F1421	CGCTTAGCACAAAGTGGTGA	20	CCACCCATAAAATCCCACAC	20	173
F1422	CTGGTCTTCGAGCTCGTCTT	20	CATTCCCAAACATATTCCCG	20	215
F1423	GCCTCCTTAAGGCTACACCC	20	CAAGCAAACCACGAGAGTCA	20	192
F1424	AGTACCAACCCAGCATCAGG	20	AAGGACAGGCCTATTTATTGTTTG	24	274
F1425	GGTGAGATTGGAGTTGCCAT	20	CATTCCCAAACATATTCCCG	20	269
F1426	GCTCAGATCCCCATCAGTGT	20	CTCTGACACTCTTACCCGCC	20	293
F1427	AAAACATGACGGGGTCTGTC	20	ACGGAAGATTGTGTTGGTCC	20	238
F1428	AGGAGGGTCGTAGCAAGGAT	20	TGAAAAACTTCCTCGCCAAC	20	236
F1429	AATATCCCTTTTGTCGCACG	20	ATGCATTGATGGGCTTGATT	20	181
F1430	TTTAACTAGCCGTGCATCCC	20	CGTCGTTCCTCCACTCATTT	20	230
F1551	TGCATGTCGCACACTGAATA	20	ACGCAGGAGAACTGCGTATC	20	149
F1552	ACCCTACTTGCGGGAAAAGT	20	TATAGCGCAAGGCACAACTG	20	178
F1553	ATTTGACCTGTGACCTCGCT	20	AGGGCTCTCGAGGAGTGTTT	20	195
F1554	CCTGAACGGAGAGTCGTAGC	20	GTGGAGCAAGAAAGGAGCTG	20	253
F1555	AGCATTCTCCATGCGTCTTT	20	GGTTTAGTTGCCTACTATGGTGGT	24	191
F1556	TTGTCGGCATGGTTCTGTAA	20	TCTCGGACGAAAAGGACAAT	20	212
F1557	CATTGGACGAGTTACGGCTT	20	GATTTTCAACACATGCACGG	20	255
F1558	ACTAGCACTTGGGCATGGTT	20	GAAACAATGGAGTGGGGTTG	20	157
F1559	GGAAGGCCATGTGTTCAGTT	20	CGGACTGAAACCAGTTCACA	20	215
F1560	AGAATCCCGAGACAAACTCG	20	CCCTCCAGCAGCTCTACACT	20	155
F1561	GCCATTGCCACTGATCTCTT	20	CGTTCTCCGTTGAGAGGAAG	20	195
F1562	CCAAAAGAGAGTGGCAGGAG	20	AAGGACCGTCCCCTTGTACT	20	140
F1563	CGGCCTACTGCCTAGTCATC	20	TTGAGGTGTCAACGGATTCA	20	193
F1564	AGAAACGCAGAGAGAGCGAG	20	TTGGTCAGCAAAGGACACAC	20	182

Table S2. *Cont.*

Primer Name	Forward Primer 1 (5′–3′) Seq	Size	Reverse Primer 1 (5′–3′) Seq	Size	Length
F1565	GAATCCTGGCCTTGTTCTCA	20	TGGCCTGACGAGGAGAATAG	20	283
F1566	CGAGAAAACTCTGTCTCACACAC	23	GATTGGTTCTTCTCGTTGCC	20	143
F1567	CAATATCTCGTAAGGTACTCATGTGG	26	TAACCCAAAGATTTGACGCC	20	223
F1568	ATGCGTAGGAGGACATGAGC	20	TAGTAGGAGCCAGGAGGTGG	20	218
F1569	CGGCCAGTGAGGAGTTACAT	20	CAGGAGAGCTGCGTGTCAT	19	233
F1570	TCATTTCGACCATGCAAATA	20	TCACACACTCGCACACACAC	20	249
F1601	GACTTTTGCTGGCAAGTTCC	20	TGAATCTCCAAGCTGCAATG	20	158
F1602	TACGCGGGGTCTGTAGTAGG	20	GGGACGTCTCTCAAGCCAC	19	144
F1603	ATTGGACGAGTTACGGCTTG	20	TAGCACACGGCACAACTAGC	20	120
F1604	GTCCGTAGGTCGTCCCTTGT	20	CAAACAACACATACACACAACTCA	24	215
F1605	TGAAAAACAAGCCCGAGTTC	20	AGAGTTCACGACGCCTTCTC	20	265
F1606	AGCCACCGCTAGAATGCTTA	20	CCCACTCCACTCCTCGTCTA	20	179
F1607	GACGTGAGACCTGTCTTGGG	20	TCACCTACGCTCTTGTGACG	20	167
F1608	CACACGGGGTTTAGACTGGT	20	ACTAAAAGGAAGAACCGGGG	20	246
F1609	GGCATGTTATTTGCAAGGTG	20	GCCTTTGCGTTGCTACAGAT	20	219
F1610	GACCCAGCGATCAGTCTCTC	20	CTCTTGTCGTCTTGGTCCGT	20	205
F1611	TTCTACCCCCTCGTTTGTTG	20	TGCGGTATTGTGATCGAAGA	20	226
F1612	ACGAATTACGGCTTGGTCAC	20	TTATTCGCTATCGGACTCCC	20	157
F1613	AGGCAAGTCATCCTTGGCTA	20	CGTTCTCCGTTGAGAGGAAG	20	243
F1614	AGCCACCGCTAGAATGCTTA	20	CCTCGTCTACACATCCTTTTCC	22	169
F1615	CTGATGCAGCTGGTGCTACT	20	CAGTCAGCAAGATGCAACGTA	21	150
F1616	TTCTACCCCCTCGTTTGTTG	20	ATTGTGATCGAAGACTCGGG	20	224
F1617	GCATTGGACGAGTTACGGAT	20	TTATTCGCTATCGGACTCCC	20	156
F1618	AAAAATTTGGCCTCACATGC	20	TACAGACTGGTCCTGGCCCT	20	217
F1619	CGCTGGAATGCTTAGGAAAT	20	TTTCCTTTTCGTAACCCACG	20	157
F1620	CTGATGCAGCTGGTGCTACT	20	CAGTCAGCAAGATGCAAACG	20	152
F1621	AAAAATTTGGCCTCACATGC	20	TACAGACTGGTCTGGCCCTT	20	218
F1622	GGTCATGTGAGTCGATAAAGCA	22	TGGGTAAACGGACAGTTGTG	20	168
F1623	ACTACGTCAGCCCCATGAAC	20	TCCTTTACCTACCTACGGACG	21	247

Table S2. *Cont.*

Primer Name	Forward Primer 1 (5′–3′) Seq	Size	Reverse Primer 1 (5′–3′) Seq	Size	Length
F1624	CTGATGCAGCTGGTGCTACT	20	CAGCAAGAATAGCAACGACTAAGA	24	153
F1625	ACCCAACCGTATATCCAACG	20	TGTCACAGTTGTCCTGGCAT	20	274
F1626	ATTGGACGAGTTACGGCTTG	20	ACGACGACGACGTACCTACC	20	154
F1627	GTTCCAAGGACGTGTGTGTG	20	GTCGTACGGTTGTCGTCGTA	20	131
F1628	CATTGGCCGCCATAGAGTAT	20	AACTAATAGTACGTACGACGCGA	23	158
F1629	ATAGGCAGGTGGTCGATACG	20	CGACGTACTTACGGTTCGTACTACT	25	221
F1630	TAGGAGTTCTCGCACCGACT	20	CTAACCGTAACCGTACCGGA	20	194
F1671	CGCACCGGTACTAAGGTGAT	20	TAGTAACCGAGCAAGGGGTG	20	288
F1672	ATTTGACCTGTGACCTCGCT	20	CCTTTCTGTTTCTGCAAGCC	20	215
F1673	AGCATTCTCCATGCGTCTTT	20	AGCACATAAAGCCTCCCTGA	20	263
F1674	TCATGGCAGCACACTCTTTC	20	CCAATCACTAACCACAAGACCA	22	117
F1675	CTGGGTGTAGAATATCGCGG	20	ACATCCTCAGCCCAATCAAC	20	283
F1676	CTCGATCGATCAGGTTTTGG	20	GCTCGACTTGGCGATTAAGA	20	249
F1677	GGTGCAACAAACCTAGCTCC	20	TCCCAAAATCCCTCATCAAG	20	190
F1678	ACCCTCCAGCTTTCCATCTT	20	TCTGAAGAATACACACGCGG	20	230
F1679	GGGGGTACGGACATAGTTGA	20	GAGCGCGAGAAGAAAGAAGA	20	199
F1680	CACATTTCAAATAACCTTCGTCTC	24	TGTTGTTCGGGAGGAATAGG	20	288
F1701	TGTTTCGGTCCCAACCTTAG	20	GAGGGTGAAGAGATTAGGGAGA	22	241
F1702	GCTTAGCTTCTGTCGGATCG	20	TGATCTAGCCCCATCAGGTC	20	174
F1703	CAACAAGGTTGGTTGGCTTT	20	ATGCTGCTGCAGATGTTTTG	20	166
F1704	GGCCTGGTATTTGTAGCAGC	20	CCAGTGAATCCCCTGTTGTT	20	227
F1705	CGATCCTACTACCTACCGGC	20	AAAGACGAAGGAAGACAGCG	20	147
F1706	ATCCTCCATTGCTGTCCAAG	20	AAGTGCCTTGTGTCCTTTGG	20	157
F1707	ATTGCGGCAACCTTTTTATG	20	GTCTTCGAGTGAGAGGACCG	20	148
F1708	ATCCTCGAGAGCATGAAGGA	20	GGGTAGTAGGTAGGGAGCCG	20	259
F1709	CTTCGACTCCACTTGCCTTC	20	GCAGCTAGGATGCTTGGAAT	20	170
F1710	GTTCTGCTCTGCTTGGCTCT	20	CTCCCCTGATTATTGGCAGA	20	214
F1751	CCAGCTTATCAGCCACTGGT	20	GGGCGTTGTTGTTCTATCGT	20	261

Table S2. *Cont.*

Primer Name	Forward Primer 1 (5′–3′) Seq	Size	Reverse Primer 1 (5′–3′) Seq	Size	Length
F1752	GCGTGAAGTAGCGATGAGGT	20	ACGCCGTGCACAATACTTTC	20	234
F1753	CCCAATGCAAGTGAGATGTG	20	TTCTCCAAAATTAGCGGTGG	20	219
F1754	TTCGATGGCTAGCACACAAG	20	TTTCTCTGCCTCTCGAATCC	20	214
F1755	ATCAGTCACGCACAGGTTTG	20	TCGTTGACCACCTTTCTCCT	20	147
F1756	CGACGACCAAATTTTGCTTT	20	TTGCTGTTTGTCATTCCCAG	20	160
F1757	TTCGATGGCTAGCACACAAG	20	TTTCTCTGCCTCTCGAATCC	20	218
F1758	TGTTTCTATCTGGCCCCTTG	20	CGATCCCTCGCAAGGTTAC	19	161
F1759	TCAGGTCTCTGAACATCCCC	20	GGTAGCCAGGATCAGCCAT	19	158
F1760	TGTGGGAGAGAAGTGGGC	18	CAAGGAAGGAATAAACCGCA	20	187
F1761	GAGATGGTGCGGATTCTGAG	20	TCATTTCCACTGTCACTGCC	20	146
F1762	TTGCAAGTAAGGTCGAAATTGA	22	CACGTCAACCAAAATACGCA	20	242
F1763	TCAGGTCTCTGAACATCCCC	20	GTGTAGCCAGGATCAGCCAT	20	158
F1764	CTGAGGTGTCTCTTGCCCTC	20	GTCCCACTGCGTTGAAAAAC	20	158
F1765	TTTCTATATTGTAACGACCGTGGA	24	TGATGAGAGCATCGTCGTTC	20	237
F1766	AAGATGAACAGAACCGCAGG	20	AACAACGCTGCAACAACAAC	20	163
F1767	TTGGGTTGTGTGCTTCATGT	20	CTCCTCCTCCTCTCGAGGTC	20	149
F1768	TGCTCATTGCACACACTACG	20	TGTTTTGTTTGGTTCGTTCG	20	155
F1769	AAGAGTCCTTCAACTGCCCA	20	CGTAGTACGTCGCACGAAAG	20	222
F1770	CTCGATCGCCTTTCTTCCTT	20	GTACGTACGACCGACCGA	18	152
F1901	TGCTCTAGTTGCAGTGGTGC	20	AGTCGTTTCTACCCCTCCGT	20	275
F1902	AGGGTAAACAAGAACGCACG	20	ATGTCTCCTCGTTATTGCGG	20	236
F1903	AGTTAACCCTTGTGATGCCC	20	TCTGGTTCATGCGTCTAACTG	21	217
F1904	TTAACCGAACAAGGAGTGGG	20	AAACCTAATCCGGTAGCCGT	20	240
F1905	TTAACCGAACAAGGAGTGGG	20	ATTGGACGAGTTACGGCTTG	20	206
F1906	GAGACGCCTTTCTCAACCAG	20	GATTCGATTTGCTCGCTTTC	20	205
F1907	AGCTGATATCTCAGGACGGC	20	CAATTAGGTTCGTAACGGTAGAGG	24	252
F1908	AGTTAACCCTTGTGATGCCC	20	CGTTGTTGGTCCTTCTGGTT	20	233
F1909	TGCAACTTCGAGTTGTTTGG	20	TTGGTAACGTCCACGTGTGT	20	246

Table S2. *Cont.*

Primer Name	Forward Primer 1 (5′–3′) Seq	Size	Reverse Primer 1 (5′–3′) Seq	Size	Length
F1910	CATGCATAGGCTCGTTTTCA	20	GAGTGGAGTGGTTACGGGTG	20	241
F1931	TCCAAGAAAGGGAAAAACGA	20	CGAGTTCTTCTCAGGGTTGC	20	285
F1932	TGGCGCTAACCTCTTGTTCT	20	ATTGTCCTATCACTTGCGCC	20	291
F1933	TGCTTCACGGATGTTCTCTG	20	GCAGATCAAACCAATAGGGC	20	232
F1934	TCTGCTCTTCAAATGGGCTT	20	GTGTGCATCTCTCCGAGTCA	20	208
F1935	GATTTTCAACACATGCACGG	20	GTCAAAGGCATTGGACGAGT	20	261
F1936	ACACATGCAGGGGATCATTT	20	ATTGGACGAGTTACGGCTTG	20	238
F1937	CTAGAGACTTTCATCGCCGC	20	ATAAGCAACCGAGCAGAGGA	20	242
F1938	ATTTGGTGCCCAAGAAATGA	20	ACCAGCAGCCATTGAATCTC	20	200
F1939	CATAGCTCACGGCACAACTG	20	TATGGTGGGTAATGGCGTTT	20	245
F1940	GCAGTGGGTCAGCTTATGGT	20	TCTCTCTGTGTGTGTGCGTG	20	210
F2011	ACACATGCAGGGGATCATTT	20	ACGAATTACGGCTTGGTCAC	20	234
F2012	TACAGGGGATCATTTTCGGA	20	ACGAATTACGGCTTGGTCAC	20	236
F2013	CATGTAGCTCAGCTCCCCTC	20	ATAGTCCACTGCAAGATCAGATT	23	210
F2014	AGGTCCATCAACATTCCTGC	20	GTTGGTGGTCGGTCTTTGTT	20	280
F2015	TCGTTTTCGAAAGGGTTTTG	20	GCATTGGATGAGTTACGGCT	20	223
F2016	AAGGATCCCCAGTCGATTTC	20	TTTCACTTGCGTCGTAGTCG	20	218
F2017	TCTACACAGCCCTACTCGCC	20	GCTTGGTCTGAGAGGAGTGG	20	251
F2018	CTTTGTGGCCTTTCATCGAG	20	CTGCTTCCTCTTTCCCCTTT	20	216
F2019	CCTCTCCTTACACGGGGATT	20	TTGATTATGCTTTGGAGGGG	20	242
F2020	CTGGTCCGAGTGGAAGAGAG	20	CTCAGTCAGCCCGTCCATAC	20	233
F2061	GGGATCGTTTTTGGAAAGGT	20	GTCACGGAATTGGACGAGTT	20	248
F2062	CGTTCTCCGTTGAGAGGAAG	20	CCTATCGATTGTCCATTGCC	20	206
F2063	GCAGTAGTTAGGGCAGGCAC	20	GGCCTAGTAGCTGCTGGATG	20	210
F2064	TGAAAAACGAAGGGGATCTG	20	GATGCGTGGGTGTGGTGT	18	227
F2065	TTAAAGCAACACCCGACCTT	20	GCGTTTGGAATTTTGTAATCA	21	201
F2066	AATCCCATCAACTGTCGCTC	20	GCTAGCAATGCTGTCATGGA	20	216
F2067	CGTTCTCCGTTGAGAGGAAG	20	CAGGCGTCCTACTCGATTGT	20	222

Table S2. *Cont.*

Primer Name	Forward Primer 1 (5′–3′) Seq	Size	Reverse Primer 1 (5′–3′) Seq	Size	Length
F2068	TGAGATTGGCATCAAGCAAG	20	TTTCTGGTCAGTTCGGTCAG	20	287
F2069	AGCGATATGTCCTCCAATGC	20	GGTAGTCGTTCTTGTCTATCTTTGT	25	240
F2070	CTCCTCCTCATAAAACCCCC	20	GTCTCCTCCCTCCCTCCTC	19	213
F2071	TGGAGAGAAAGTCAATGGGG	20	AGTACATTGTCGGGAGTCGG	20	223
F2072	TGCTTCATGTGCATGGATTC	20	CCACTCCCTTCTCGGAAAGT	20	236
F2073	TGGAGAGAAAGTCAATGGGG	20	GAGTCGGTTGTTGAGGTGGT	20	216
F2074	GCCACACTAAATAAGCTTTGTGTC	24	TGGTCGTCACTGATTACGGA	20	236
F2075	AAACGATTTGTCACGGGAAG	20	AGGGGGACGAACTTCTTCAT	20	263
F2076	CGAAGCATGTTACTCCCCAT	20	AACATAGGTGGGTGGTGGG	19	225
F2077	AATTGTGCAGATTCCGATCC	20	TTTGTCGAGCAACTGGAAGA	20	224
F2078	GCATGGGCTTGTTTTCAAAT	20	CATTTGGGGTAGTGGAGTGG	20	240
F2079	ATGACGAAGCCTCTGAGCAT	20	AGACATGCGCAAACTAGCAC	20	221
F2080	CGGGAAGCAAATGTTCAGAC	20	ACTTCTTCATCTGCACCGCT	20	241
F2181	GAAAAGGACTGACAGAGCCG	20	CCCTCCGTCGTTTCCCTT	18	244
F2182	CAGTTCAGTGCAGTGCAGGT	20	CTTCTTCCTTCCTTCCTACCTC	22	229
F2183	AAATCGACGAACGACTCACC	20	CGCCGACTCTCTCTCTGTCT	20	200
F2184	TGTTCGAAAGCCAAGAATCC	20	TGGAATCGCGGAATACAAAT	20	247
F2185	GAGTTAGAGGACAGCGTGGC	20	TGCAGCAGAGAATGTGCTACT	21	210
F2186	TAGCCCGTAGTGCTCCAACT	20	CACAAGATGGCACGGTTCTA	20	214
F2187	CGATTGCACACACGGATAAC	20	GTCGTTGTGCTTGCACTTGT	20	289
F2188	TTGATGGGATCAAGAGGAGG	20	AGCAGCCGACGTGTTTTACT	20	242
F2189	ATTCCATGGCACTGGTTCTC	20	CCTCTCTCTACTTTTCTTCGTCA	23	252
F2190	ACCATGAACAAAAAGCAGGG	20	CTTCTCCTTCCCGGACCTC	19	217
F2201	CCACCTCGGAGAGGTTGATA	20	TCTGACTCCACCGATTTTCC	20	279
F2202	GTCCTCACCTCCTTTTGGGT	20	CCTTCGTTTCTCTCTCGTCG	20	250
F2203	ACGGAAGGAAGAAAGGTGGT	20	GGCCCTCGCCTTACTTAGTC	20	206
F2204	CACCACACCACCTACGTGAC	20	GATAGGGGATCCGGAGTCTG	20	240
F2205	AAGCAGTTGCAGCAGTAGCA	20	TCGTCTCTCGTTAGGTTCGG	20	207

Table S2. *Cont.*

Primer Name	Forward Primer 1 (5′–3′) Seq	Size	Reverse Primer 1 (5′–3′) Seq	Size	Length
F2206	GGCCTAACGGGAGTTAAAGG	20	TGCAGCAGAGAATGTGCTACT	21	230
F2207	GGTTACTCCTGCTGGCTCTG	20	CGTTCGTCTTCCTTCTCGTC	20	215
F2208	CACGACAGTCGAGTTCCTCA	20	GCTTGTCCACGCAAGTACG	19	252
F2209	GGAACCAAGAAAACCCCAAT	20	GTACGTCGAACGAAGACGAC	20	236
F2210	CTTCGCTCGTTTTCAACTCC	20	GACTGCAGAGCACGGGAC	18	206
F2281	TTCTCCGTCAGCTCACATTG	20	TCCATTGTTCATTTAGTAGAAACCT	25	251
F2282	ATTGGACGAGTTACGGCTTG	20	TATGCCCACGAGTCAATCTG	20	224
F2283	CGTGTTGCGGTACCCTACTT	20	TCCATGCACAACTAGCTGGA	20	205
F2284	CTACGGAAGAGAGTGGCAGG	20	TATCCACCAACGTGGGATTT	20	221
F2285	TCTTGTTCTGTACAGTTTGTATGGG	25	ACCATTGGCCGATATTACCA	20	211
F2286	GCTAATCACGCGATCAGACA	20	TCCCTTCGTACCCCTTTCC	19	226
F2287	CGTGTTGCGGTACCCTACTT	20	TAGCTCACGGCACAACTAGC	20	210
F2288	GACCCAGCGATCAGTCTCTC	20	CTCTTGTCGTCTTGGTCCGT	20	201
F2289	CCAACATAGTGTCATTGCGTG	21	GAGGCCCGTTCGTTATGTC	19	243
F2290	GACCCAGCGATCAGTCTCTC	20	GACCTCACCTCTTCGTCGTC	20	215
F2301	GCCATCGATCACCAGCTTAT	20	CGGACGACGACTAGCAGAC	19	204
F2302	ATTCTCAGGGTGTGCGTGAT	20	GGGCGACGATACCCTACTAC	20	265
F2303	GCATGCCCTGTCATGTATTG	20	CACACAAGAGCGTGGAGGTA	20	242
F2304	GCGGTACCCTACTTGTTGGA	20	TCCACTAGCTCACGCACAAC	20	221
F2305	CGCACTAGCCCTTGTCTTTC	20	CGCCCTACGAACAAATCACT	20	225
F2306	TAGTTGATGCGTCTCGTGCT	20	GTCGGCGAGACTCCCTACTC	20	237
F2307	AGCTGTGGAAGAACGGAAGA	20	TCAGAACTAGCGCAACAATGA	21	224
F2308	AAAAATTTGGCCTCACATGC	20	CCGGTTTACGGTACACGACT	20	239
F2309	AAAAATTTGGCCTCACATGC	20	CGGTTAACGTACGAACTACGG	21	245
F2310	CCGCATATGCATACGTCAAG	20	CCGACTGTCTAGGTCGTGCT	20	215
F2361	TCACGCTTTCCCTTATTTGG	20	TCGGTTTTTAGCCACGAGTC	20	194
F2362	CCATTCCAGCAGATTTCGAT	20	CAGGTTGGAAGCATAGGTGG	20	220
F2363	GACCCCCGCCTTTTTAGTAG	20	TGTTACGATGATGCGGCTTA	20	202

Table S2. *Cont.*

Primer Name	Forward Primer 1 (5′–3′) Seq	Size	Reverse Primer 1 (5′–3′) Seq	Size	Length
F2364	CTTCTTGGATGGAAGCGAAG	20	ACCTGTTCTTGCCATTACCG	20	277
F2365	GTGGAGCTGCGGTACTTTTC	20	CAGCACATGAAAAGAAGAGATAGA	24	252
F2366	CGCTTCTATCACCTCTTCCG	20	GCATTCCTTTATAGCTGCGG	20	260
F2367	TCTCTGGGAAGTTGTTCAGGA	21	TGACGCGGTTAGTCAAAATG	20	207
F2368	TCACGCACTACCTCTCTCTCC	21	TGAGTGATTGTGTGTGAGGG	20	190
F2369	AGAGAGGGGGCTGGTTGTAT	20	AACCATACGGAGGACACGAG	20	203
F2370	CCAAGTCCCAAGGTTTTTCA	20	GAGAGAGAGAGGCGTGTGCT	20	252
F2381	TGTGTGTCCGTGTCTGTGTC	20	TGCTTGATTCTTGAAGGTGG	20	295
F2382	CAACAAGGTTGGTTGGCTTT	20	ATGCTGCTGCAGATGTTTTG	20	165
F2383	CCCCTACTCCCGTGAAACTT	20	TGTCAACGTATGCGTGTGTG	20	162
F2384	GACGGTAGCCATGGTCTGAT	20	ATCCAGAAGCCGGGTAAGTT	20	230
F2385	GTCAACTCATCATCCCACCC	20	CATGTGTCGTCACTTTTGGG	20	279
F2386	GGCTCCACTTCCTCCTCTTG	20	TCTCGGCCAACAGAAACTCT	20	253
F2387	GAAAGCTGGCTGAACAGGAC	20	AGTTTGATCATAGGCCACCG	20	196
F2388	GATAGCCTGCCTGATTGGTG	20	AAGGCTTAGGCCCATCAAGT	20	263
F2389	TCCACAGTCAGAGTTGGCAG	20	AAATACATGTCACTCGCCCC	20	236
F2390	CCCAATGCAAGTGAGATGTG	20	GGGGTTTCGATCTGGATTCT	20	227
F2531	TTGTTCTCTGCAGGTCGATG	20	TCTCACGGTTACGTGTCCAG	20	247
F2532	AATGTCACAGGTTTCCCTCG	20	GCTGACGCTTGTGTTGTGTT	20	177
F2533	AAGGCTTAGGCCCATCAAGT	20	GATAGCCTGCCTGATTGGTG	20	278
F2534	AAATCTTCACACCCAGCACC	20	ACCTTGTATTGCACCTTGCC	20	257
F2535	TCATTCCACCAACGAATTGA	20	AATCTTCGAACGCGACTTGT	20	188
F2536	ACCATTGGCCGATATTACCA	20	AAAAGATTGGCTCGTGGTTG	20	267
F2537	TACTCCAGTGTTGTTGGGCA	20	TCTTTGGTGCAGAAGATGGA	20	196
F2538	CAGTGTGAGCTCGAAGATGC	20	ACGGAAGGGAACGACTGAC	19	235
F2539	TGAATCTCCAAGCTGCAATG	20	CCGTGGAAATGGTCCTCTAC	20	197
F2540	TATTCGAGCCCCATTTCTTG	20	GCGTTATCCGGATGATGAAG	20	184
F2551	TATTCGAGCCCCATTTCTTG	20	AGCGTATCCGGATGATGAAG	20	185

Table S2. *Cont.*

Primer Name	Forward Primer 1 (5′–3′) Seq	Size	Reverse Primer 1 (5′–3′) Seq	Size	Length
F2552	AAACACAAGCCGAATTGTCC	20	CTGAGGTGTCTCTTGCCCTC	20	190
F2553	TTAACCGAACAAGGAGTGGG	20	ATTGGACGAGTTACGGCTTG	20	198
F2554	GCCCAAAAACTCATTCTCCA	20	GCACATATGTTTGTGCAGGG	20	171
F2555	GGGAACACGAACACATGGTA	20	TGTATGTCTCCTGTTATGCGG	21	176
F2556	AAAGCAAGAAGGTCCGAGGT	20	CGGCGGCAATTATCTTGTAT	20	237
F2557	AAAAGGCAGCTGAGCATCAT	20	GCCCTTTCGTCTCTGTTTTG	20	192
F2558	GAGACGCCTTTCTCAACCAG	20	TGTCTCGTCTTCTCCGATCC	20	186
F2559	TGCTCTAGTTGCAGTGGTGC	20	GGAGGGCATTGTACTGGAGA	20	162
F2560	GAAGGTAGCCACCCTCTTCC	20	GTCTCTTGGGTGTGCACTGA	20	295
F2731	CGTTCTCCGTTGAGAGGAAG	20	AGGCAAGTCATCCTTGGCTA	20	235
F2732	TCCAAAACATTGAGTGGGGT	20	ATTGGACGAGTTACGGCTTG	20	194
F2733	ACCCCATCCTTTCTCCAAAT	20	TCTCCTCACTTTCGATGGCT	20	288
F2734	CACACAGATATTTGGCACCG	20	TGAGGATCCGAAAAGATTGG	20	216
F2735	GCATGGGCTTGTTTTCAAAT	20	ATTGGACGAGTTACGGCTTG	20	256
F2736	CTCTATCGACCGCATGTGTG	20	CCAGCTTATCAGCCACTGGT	20	162
F2737	TTTGGTTGGTTGGATGGATT	20	TCGTAGGGTGAAGCGACTCT	20	196
F2738	GCAGTTTTGTTTCGATCGGT	20	GCATGGTTTGTACACTTGCG	20	275
F2739	ATACCCGAAGCATTGAGTGG	20	TGTTTGGGTCGAAGAATAGTCA	22	171
F2740	AGCTTTGGCATGGGATTATG	20	AACAGGTAGTCATGCGTGTGTC	22	182
F2781	ACGTAGCATTTTCGGTTTGG	20	GGATTTGTGCCGTGTATGTG	20	298
F2782	GCCGGAGTATAGATCCGACA	20	GTCAGGCCGTGAACGTTATT	20	175
F2901	ATGCACGCACGAACACATA	19	TCTTGATCATCACCAGCACC	20	280
F2902	ATTGTGCTCACCTACGCCTC	20	GCTACTCCCATAGCCCCTTC	20	198
F2979	CACCATGTGTATGCGTGTGA	20	GGAGAGGAGCTTTCAGAACCA	21	234
BM114	ATCGTAGAAACCATTGGCCC	20	TGACCCATGGACACTTTTCA	20	279
BM136	AATGTCACAGGTTTCCCTCG	20	GCGAGAAAGAGGAGAGGGTT	20	225
BM212	TATAGCCTCACCGCTCGTCT	20	GGCCTGAAAACTCAAATGGA	20	206
BM289	TGGGACAATATGGCAAGGTT	20	ACAAATGCCTGATGGTAGGC	20	237

Table S2. *Cont.*

Primer Name	Forward Primer 1 (5′–3′) Seq	Size	Reverse Primer 1 (5′–3′) Seq	Size	Length
BM295	CACACAGATATTTGGCACCG	20	TGAGGATCCGAAAAGATTGG	20	214
BM306	ATTTTCTGGGCAATTCAACG	20	GTCCTCATCCCTTCCCTCTC	20	191
BM341	GTGAAGAACTCTCGATCGGC	20	ACTGGGTAGTACACGGCGAG	20	305
BM344	AGCACTGAGGCACAATTCCT	20	GTGCTGGGGTTTGTGACTTT	20	231
BM374	CTACCGCTTCAAAACGAAGG	20	TGTCCCACTCTCCTACCTACTACC	24	178
BM375	CCAGCGTTAATCATCTCGGT	20	CACCCACTTCGACCCAAC	18	202
BM377	CGAGCAGCAGTTCTCAAGTG	20	TCCTTCGGTTCGTTCTATGC	20	171
BM378	ATGGGATGCACAGGTACACA	20	TCCTTAGGTCATCGTCCTATTTG	23	260
BM379	ACAGAATGGCTACCTCCCCT	20	GGGCGTAACCGTTAAACCTA	20	214
BM395	AGGCGGCTTAAAAATCCTGT	20	TCTCCATGCAAGCAATCAAG	20	239
BM396	TTGATTATGCTTTGGAGGGG	20	CCTCTCCTTACACGGGGATT	20	248
BM397	TTCCGGTTACAGACGGTAGG	20	TCTTGTGACGTTCTGCCAAG	20	194
BM411	GACCCAGCGATCAGTCTCTC	20	TTGTCGTCTTGTCCGTCG	18	198
BM412	AAAAATTTGGCCTCACATGC	20	CCGGTTTCGTACAGACTGGT	20	233
BM481	TATATGGGCTGCACGAGGTT	20	TTCTCTGCAACGACATACGC	20	264
BM482	CTGCCTTCTCCTTGCAGTTG	20	GGTTAAGAGCCCCCTTTTTG	20	259
BM483	GACCAGAGACTTGGGCTTTG	20	TCACTCACTCACTCATCCGC	20	243
BM484	GGACAGGATGAGGAGGATCA	20	CGGTTACCATCGCCTTCTTA	20	235
